# Functional Genomic Screening During Somatic Cell Reprogramming Identifies DKK3 as a Roadblock of Organ Regeneration

**DOI:** 10.1002/advs.202100626

**Published:** 2021-05-13

**Authors:** Frank Arnold, Pallavi U Mahaddalkar, Johann M. Kraus, Xiaowei Zhong, Wendy Bergmann, Dharini Srinivasan, Johann Gout, Elodie Roger, Alica K. Beutel, Eugen Zizer, Umesh Tharehalli, Nora Daiss, Ronan Russell, Lukas Perkhofer, Rupert Oellinger, Qiong Lin, Ninel Azoitei, Frank‐Ulrich Weiss, Markus M. Lerch, Stefan Liebau, Sarah‐Fee Katz, André Lechel, Roland Rad, Thomas Seufferlein, Hans A. Kestler, Michael Ott, Amar Deep Sharma, Patrick C. Hermann, Alexander Kleger

**Affiliations:** ^1^ Department of Internal Medicine I University Hospital Ulm Albert‐Einstein Allee 23 89081 Ulm Germany; ^2^ Institute for Diabetes and Regeneration Helmholtz Zentrum München Ingolstädter Landstraße 1 85764 Neuherberg Germany; ^3^ Institute of Medical Systems Biology Ulm University Albert‐Einstein Allee 11 89081 Ulm Germany; ^4^ Department of Gastroenterology Hepatology and Endocrinology Hannover Medical School Feodor‐Lynen‐Str. 7 30625 Hannover Germany; ^5^ Core Facility for Cell Sorting and Cell Analysis University Medical Center Rostock Schillingallee 70 18057 Rostock Germany; ^6^ Diabetes Center University of California San Francisco CA 94143 USA; ^7^ Institute of Molecular Oncology and Functional Genomics TranslaTUM Cancer Center Technical University of Munich Ismaninger Str. 22 81675 Munich Germany; ^8^ Bayer AG Research & Development Pharmaceuticals Müllerstraße 178 13353 Berlin Germany; ^9^ Department of Medicine A University Medicine Greifswald Ferdinand‐Sauerbruch‐Straße 17475 Greifswald Germany; ^10^ Klinikum der Ludwig‐Maximilians‐Universität München‐Großhadern Marchioninistraße 15 81377 München Germany; ^11^ Institute of Neuroanatomy & Developmental Biology INDB Eberhard Karls University Tübingen Österbergstr. 3 72074 Tübingen Germany

**Keywords:** functional shRNA screen, regeneration, reprogramming, Wnt‐/Hedgehog‐signaling

## Abstract

Somatic cell reprogramming and tissue repair share relevant factors and molecular programs. Here, Dickkopf‐3 (DKK3) is identified as novel factor for organ regeneration using combined transcription‐factor‐induced reprogramming and RNA‐interference techniques. Loss of *Dkk3* enhances the generation of induced pluripotent stem cells but does not affect de novo derivation of embryonic stem cells, three‐germ‐layer differentiation or colony formation capacity of liver and pancreatic organoids. However, DKK3 expression levels in wildtype animals and serum levels in human patients are elevated upon injury. Accordingly, *Dkk3*‐null mice display less liver damage upon acute and chronic failure mediated by increased proliferation in hepatocytes and LGR5^+^ liver progenitor cell population, respectively. Similarly, recovery from experimental pancreatitis is accelerated. Regeneration onset occurs in the acinar compartment accompanied by virtually abolished canonical‐Wnt‐signaling in *Dkk3*‐null animals. This results in reduced expression of the Hedgehog repressor *Gli3* and increased Hedgehog‐signaling activity upon *Dkk3* loss. Collectively, these data reveal *Dkk3* as a key regulator of organ regeneration via a direct, previously unacknowledged link between DKK3, canonical‐Wnt‐, and Hedgehog‐signaling.

## Graphical abstract

Re‐activation of embryonic pathways is a hallmark of tissue repair after injury. During somatic reprogramming similar molecular patterns are re‐activated, thus providing an excellent platform to identify relevant factors in tissue repair. Here, Dickkopf‐3 (DKK3) is identified as key player of gastrointestinal tissue regeneration and repair via a DKK3‐mediated interplay of canonical Wnt‐ and Hedgehog‐signaling.

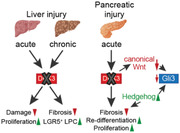

## 1 Introduction

Reprogramming of somatic cells to induced pluripotent stem cells (iPSC) upon ectopic expression of the four Yamanaka factors (OCT3/4, KLF4, SOX2, and c‐MYC) represents a tremendous advance in disease modeling, regenerative medicine, and drug development.^[^ ^1^, ^2^, ^3^ ^]^ Generation of patient‐specific iPSCs complemented with lineage‐specific differentiation can not only provide important insights into disease pathogenesis to leverage a resource for potential rescue strategies,^[^ ^4^, ^5^, ^6^, ^7^ ^]^ but can also eliminate age‐ and disease‐specific phenotypes.^[^ ^8^ ^]^ Cellular reprogramming is limited to or promoted by a variety of distinct growth factors, small molecules, transcription factors, and signaling proteins, many of which have overlapping functions in embryonic development, regeneration, tissue homeostasis, and carcinogenesis.^[^ ^9^ ^]^ Furthermore, tissue‐resident stem cells, which are closely associated in these processes, share common features with their pluripotent counterparts, such as differentiation potential and self‐renewal capabilities. Numerous signaling pathways involved in tissue homeostasis and reprogramming tightly control these stemness features. For instance, constitutively active Wnt‐signaling, a signaling pathway that improves the efficiency of reprogramming somatic cells into iPSCs,^[^ ^11^, ^12^, ^13^ ^]^ also mediates the self‐renewal of gut stem cells.^[^ ^10^ ^]^ However, the Hippo / Yes‐associated protein (YAP) and its transcriptional co‐activator with PDZ‐binding motif (TAZ) that constitute the YAP/TAZ pathway drives the regeneration of injured intestine or liver.^[^ ^14^, ^15^, ^16^ ^]^ YAP, which is expressed more strongly during somatic reprogramming,^[^ ^17^ ^]^ is directly regulated and activated by Hedgehog (Hh) signaling during liver regeneration,^[^ ^18^ ^]^ which by itself increases reprogramming efficiency.^[^ ^19^ ^]^ Both Hh‐ and Wnt‐signaling facilitate exocrine pancreatic regeneration,^[^ ^20^, ^21^ ^]^ highlighting the similarities between reprogramming and organ regeneration as well as overlapping regulatory pathways.

Functional genomic screening using RNA‐ or DNA‐interference technology such as shRNA or CRISPR/Cas9‐sgRNA libraries are established tools for the analysis of cells at the whole‐transcriptome or whole‐genome level.^[^ ^22^, ^23^ ^]^ Over the last decade, such screens were successfully employed to identify genes involved in reprogramming, in understanding disease pathogenesis, and to reveal novel therapeutic targets.^[^ ^24^, ^25^, ^26^ ^]^ However, such screening approaches were never performed during somatic reprogramming, in spite of their great potential use for the identification of factors facilitating tissue regeneration. Since numerous signaling pathways not only regulate reprogramming but also are associated in organ regeneration, we hypothesize that combining transcription factor‐induced reprogramming and RNA‐interference techniques in a functional screening approach may facilitate a highly effective platform to identify genes impairing organ regeneration. Employing this strategy, we identified Dickkopf‐3 (DKK3), a member of the Dickkopf protein family involved in Wnt‐signaling,^[^ ^27^, ^28^ ^]^ to be a limiting factor for reprogramming. As physiological tissue development occurs unhindered in *Dkk3*‐null mice,^[^ ^29^ ^]^ we assessed tissue repair upon injury postulating that similar pathways involved in somatic reprogramming^[^ ^21^, ^30^, ^31^ ^]^ also drive the regeneration of the tissue. A comprehensive set of murine injury models focusing on liver and pancreas allows us to unravel i) regulatory patterns of DKK3 upon injury, ii) DKK3 as limiting factor of pancreas and liver regeneration, iii) compartment specific effects of DKK3 loss in, for example, hepatocytes versus stem cells and acinar versus ductal cells,^[^ ^21^, ^32^, ^33^ ^]^ and iv) finally translate these data to human cohorts of acute and chronic liver/pancreatic disease models. Particularly, the 3,5‐diethoxycarbonyl‐1,4‐dihydrocollidine (DDC) diet in mice causes bile duct injury and induces ductular reaction cells expressing Leucine‐rich repeat‐containing G‐protein coupled receptor 5 (LGR5) and harboring a bipotent differentiation potential.^[^ ^34^ ^]^ Furthermore, the employment of additional chronic injury model also assists in investigating the role of DKK3 in a liver resident stem cell compartment.^[^ ^35^ ^]^ Specifically, *Dkk3* loss leverages proliferation of hepatocytes and acinar cells after acute injury and attenuates chronic liver damage by expansion of the LGR5‐positive liver progenitor pool. Mechanistically, an intimate link between *Dkk3*‐/canonical Wnt‐ and Hh‐signaling in injury‐induced tissue repair was identified operating differentially and in a time‐resolved manner with organ‐specific patterns in liver and pancreas.

## 2 Results

### 2.1 Functional shRNA Screen during iPSC Formation Reveals *Dkk3* as a Factor Limiting Stem Cell Characteristics

**Figure 1:**
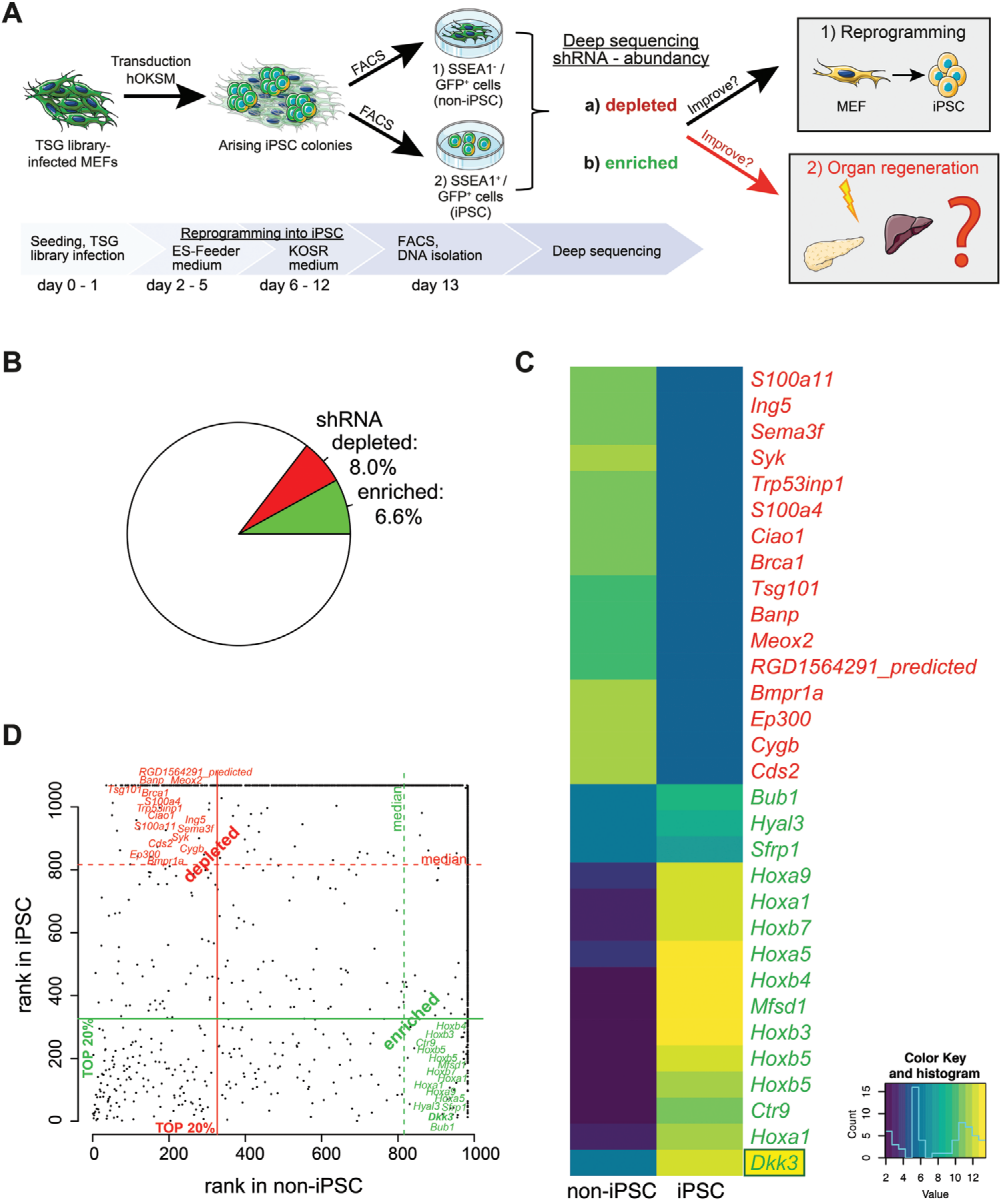
Functional shRNA screen identified factors impairing somatic reprogramming. A) Scheme illustrating the procedure of the shRNA screen with TSG library during reprogramming of MEF involving the four Yamanaka‐factors (OKSM; OCT3/4, KLF4, SOX2, and c‐MYC) into iPSC. Our hypothesis of the abundant shRNA in either reprogramming and/or organ regeneration is included. B–D) Pie chart, heatmap (log2‐transformed normalized counts), and dot plot highlighting most abundant (enriched/depleted) shRNA after reprogramming.

We applied a functional genomic screen during somatic cell reprogramming of mouse embryonic fibroblasts (MEF) into iPSCs to identify genes that are known to regulate organ regeneration (**Figure** **1**A). Since cancer‐associated genes might be crucial for stem cell fitness due to the shared molecular programs between somatic reprogramming, cancer progression, and organ regeneration,^[^ ^10^, ^11^, ^17^, ^18^, ^19^, ^21^, ^36^ ^]^ an shRNA library targeting 700 potential “cancer genes” was included.^[^ ^37^ ^]^ FACS‐based separation/sorting of reprogrammed (repro) stage‐specific embryonic antigen‐1 (SSEA1)‐positive (iPSC) and non‐reprogrammed (non‐repro) SSEA1‐negative cells (non‐iPSC) allowed retrieval of genomic DNA of distinct populations. shRNA abundance in the respective populations was determined via deep sequencing and bioinformatic analysis. 6.6% of shRNAs were found depleted in non‐iPSCs but enriched in iPSCs, while 8.0% showed the opposite pattern (Figure 1B–D). Amongst the shRNAs with highest rank in iPSCs but lowest in non‐iPSCs, we identified the gene *Dkk3* as a potential target, suggesting that the loss of *Dkk3* might facilitate somatic cell reprogramming (Figure 1C,D).

**Figure 2:**
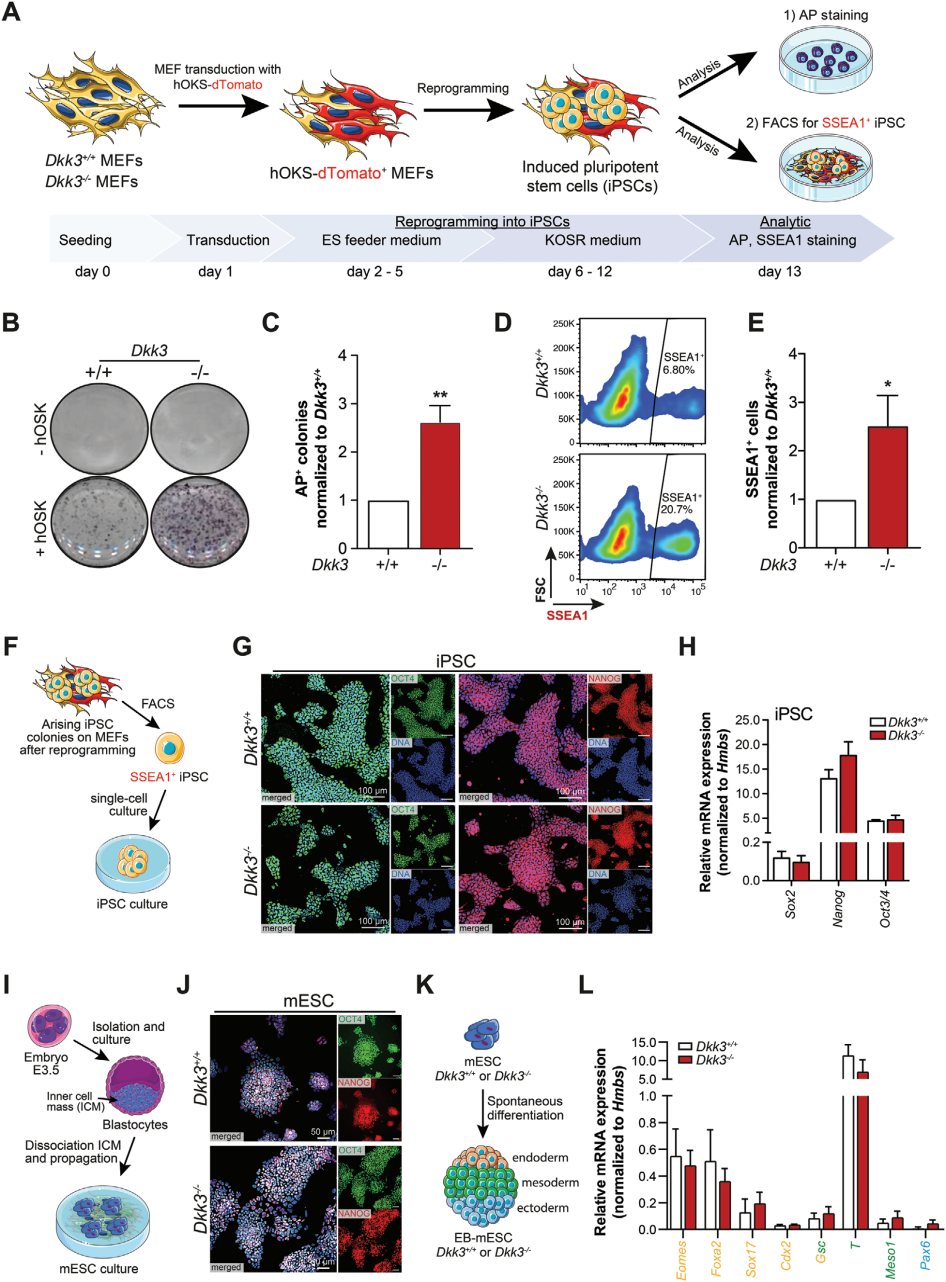
*Dkk3* loss drives reprogramming to iPSCs, but does not affect de novo derivation of embryonic stem cells and three‐germ layer differentiation. A) Schematic representation showing three‐factor lentiviral‐induced three‐factor reprogramming (OKS; OCT3/4, KLF4, SOX2). B) AP‐staining of reprogrammed *Dkk3* ^+/+^ and *Dkk3* ^−/−^ MEF into iPSC and C) the corresponding quantification of AP^+^ colonies. D) Representative FACS‐plots of SSEA1‐staining of reprogrammed *Dkk3* ^+/+^ and *Dkk3* ^−/−^ MEF with corresponding quantification of E) SSEA1^+^ cells. F) Scheme depicting single cell cultivation of SSEA1‐sorted *Dkk3* ^+/+^ and *Dkk3* ^−/−^ iPSC. G) Representative IF pictures of pluripotency markers OCT4 (green) and NANOG (red) of iPSC, and H) RT‐PCR of pluripotency marker (*Sox2*, *Nanog*, *Oct3/4*) of iPSCs. I) Illustration of the isolation and cultivation of *Dkk3* ^+/+^ and *Dkk3* ^−/−^ mESC. J) Representative IF images of mESC with the pluripotency markers OCT4 and NANOG. K) Spontaneous differentiation of mESC into EB harboring cells of the three different germ‐layers. L) RT‐PCR of endodermal (*Eomes*, *Foxa2*, *Sox17*, *Cdx2*), mesoendodermal (*Gsc*), mesodermal (*T*, *Meso1*), and ectodermal (*Pax6*) marker of *Dkk3* ^+/+^ and *Dkk3* ^−/−^ EBs. (Bar graph show mean + SEM, unpaired *t*‐test, * *p* ≤ 0.05 ** *p* < 0.01).

In contrast to other DKK proteins, DKK3 is not only a classical Wnt modulator, but also limits TGF‐*β*‐signaling during prostate morphogenesis, and maintains pancreatic cancer cells in a de‐differentiated state.^[^ ^38^, ^39^, ^40^ ^]^ However, the effect of DKK3 on iPSC formation is unclear. Thus, we quantitatively assessed the reprogramming capacity of MEFs derived from ubiquitous *Dkk3* knockout mice using three factor‐based reprogramming (OCT3/4, KLF4, SOX2 (OKS), **Figure** **2**A). Interestingly, *Dkk3* ^−/−^ MEFs formed significantly more alkaline phosphatase positive (AP^+^) colonies (Figure 2B,C) alongside with significantly more SSEA1^+^ cells (Figure 2D,E) upon reprogramming. However, single cell seeding of established SSEA1^+^ iPSCs^[^ ^41^ ^]^ from both genotypes did not differ in pluripotency marker expression, as demonstrated by immunofluorescence (IF) staining of octamer‐binding transcription factor 4 (OCT4) and NANOG, as well as by mRNA expression levels of *Oct3/4*, *Nanog*, and *Sox2* (Figure 2F–H and Figure S1A, Supporting Information). Additionally, to investigate the relevance of DKK3 for maintaining pluripotency, we derived mouse embryonic stem cells (mESC) de novo from blastocysts of the respective genotypes. No significant differences in the expression of OCT4 and NANOG (Figure 2I,J) or in the efficiency of line derivation (100% in both genotypes) were detected.

Several studies demonstrated a potential involvement of DKK3 in cell fate determination toward a specific lineage, such as osteoblast or cardiac differentiation from human mesenchymal stem cells,^[^ ^42^, ^43^, ^44^ ^]^ or the differentiation of ESCs into smooth muscle cells.^[^ ^45^ ^]^ To analyze the effects of *Dkk3* on lineage commitment, embryoid bodies (EB) from *Dkk3* ^+/+^ and *Dkk3* ^−/−^ mESCs were generated (Figure 2K). Interestingly, the expression analysis of germ layer‐specific markers^[^ ^46^, ^47^ ^]^ showed no significant differences (Figure 2L), indicating that *Dkk3* loss does not influence the differentiation toward a specific lineage. In summary, these results suggest that *Dkk3* limits the initiation of pluripotency but does not affect its maintenance, nor does it have obvious effects on the subsequent early germ layer formation.

### 2.2 *Dkk3* Loss does not Impair Colony Formation from Uninjured Pancreas and Liver

Tissue‐resident stem cells are characterized by self‐renewal and multipotency, and are essential for tissue homeostasis and promote tissue injury induced regeneration of acinar cells and hepatocytes.^[^ ^48^, ^49^, ^50^, ^51^, ^52^ ^]^ We assumed that genes driving an efficient reprogramming might also affect stem cell compartments in different organs, such as the liver and pancreas.^[^ ^13^ ^]^ Therefore, we FACS‐purified liver progenitor cells (LPCs) based on previously reported surface marker profiles.^[^ ^12^, ^13^ ^]^ We also isolated pancreatic ductal organoids (PDO), a hallmark model for adult bi‐potent pancreas progenitors maintained by the LGR5/R‐spondin axis and suitable to investigate self‐renewal capacity^[^ ^53^, ^54^ ^]^ (Figure S2A–C, Supporting Information). However, we neither detected differences in the colony forming capacity of adult LPCs (Figure S2D,E, Supporting Information), nor in the morphology, number, or size of PDOs isolated from adult *Dkk3* ^+/+^ or *Dkk3* ^−/−^ mice. (Figure S2F, Supporting Information). Conversely, after the isolation of fetal LPCs, bipotent colony formation was significantly more frequent in *Dkk3* ^−/−^ than in wildtype littermates (Figure S2G,H, Supporting Information) indicating a potential role during liver development or at least in states of higher proliferation (e.g., tissue repair). Of note, elevated DKK3 levels have been described in fetal liver and again during regeneration after partial hepatectomy of porcine liver suggesting also a potential role of DKK3 during organ regeneration.^[^ ^55^ ^]^

### 2.3 Acute and Chronic Injury of Liver and Pancreas Induces DKK3 Expression

**Figure 3:**
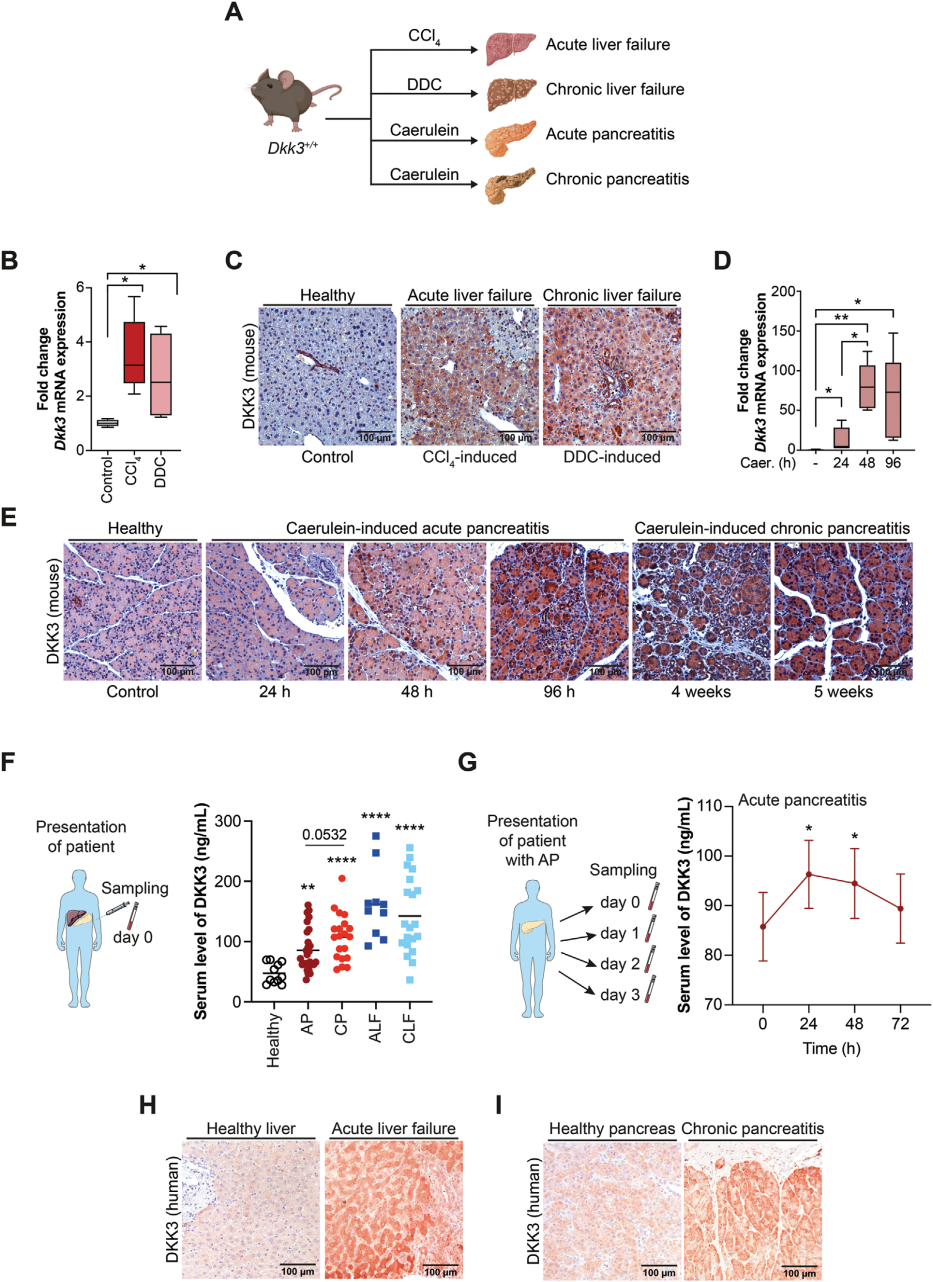
DKK3 expression is elevated during liver and pancreatic injury in human and mouse. A) Scheme shows respective mouse model to induced either ALF (CCl_4_) or CLF (DDC) and AP or CP (both caerulein). B) RT‐PCR of *Dkk3* and C) immunohistochemistry of DKK3 in control, CCl_4_ or DDC‐treated mice. D) RT‐PCR of *Dkk3* and E) immunohistochemistry of DKK3 in control or caerulein treated mice for indicated time points in respective mouse models of pancreatitis. (*n* ≥ 4 per group; Mann–Whitney U test for RT‐PCR, * *p* ≤ 0.05 ** *p* < 0.01) F) Serum levels of DKK3 measured from either healthy subject group (Healthy, *n* = 11) or patients diagnosed with ALF or CLF (ALF, *n* = 10, CLF *n* = 20), and AP or CP (AP, *n* = 27, CP, *n* = 20) at day of presentation (day 0, graph shows mean ± SEM, unpaired *t*‐test, * *p* < 0.05, *** *p* < 0.001). G) Serum levels of DKK3 of indicated time points of patients with AP (*n* = 27, graph shows mean + SEM, paired *t*‐test, * *p* < 0.05, ** *p* < 0.01). H,I) Immunohistochemistry of DKK3 in patients with ALF (H, *n* = 3) or CP (I, *n* = 3) in comparison to healthy tissue control.

To probe the relevance of DKK3 during tissue regeneration upon damage, we analyzed DKK3 expression levels in wildtype animals with chemically induced acute or chronic liver failure (ALF, CLF) or pancreatitis (**Figure** **3**A). We observed an increase of DKK3 expression levels by RT‐PCR and immunohistochemistry during the regeneration of carbon tetrachloride (CCl_4_)‐induced ALF and DDC‐induced chronic liver injury (Figure 3B,C). Induced DKK3‐expression occurred in a cell‐type and in injury‐dependent manner with a predominant induction in hepatocytes upon CCl_4_‐induced acute liver injury, while DDC‐mediated injury caused additional DKK3 induction in CK19^+^ biliary and CD44v6^+^ progenitor cells^[^ ^56^, ^57^ ^]^ (Figure 3C and Figure S2I,J, Supporting Information). Similarly, DKK3 expression was elevated in pancreata of mice with either caerulein‐induced acute or chronic pancreatitis (AP, CP) (Figure 3D,E). Intriguingly, pancreatic DKK3 induction occurred with a temporal increase (Figure 3D,E) and spatial differences as indicated by a pronounced increase within structures undergoing acinar to ductal metaplasia (ADM) (Figure S2K, Supporting Information). Of note, transient ADM formation is paramount for regeneration and repair of the exocrine pancreas.^[^ ^21^ ^]^

To translate these observations to humans, we assessed DKK3 serum levels by enzyme‐linked immunosorbent assay (ELISA) and immunohistochemistry (IHC) in patients diagnosed with either ALF or CLF and AP or CP (Figure 3F–I and Table S1, Supporting Information). Interestingly, DKK3 serum levels were significantly increased independent of acute/chronic or hepatic/pancreatic damage (Figure 3F and Table S1, Supporting Information). Similar to mouse tissue, DKK3 serum levels in patients with AP were temporally regulated with a peak at 24 h post‐diagnosis followed by a drop during recovery (Figure 3G). Immunohistochemistry of human liver biopsy specimens from patients with ALF revealed particularly elevated DKK3 levels in hepatocytes (Figure 3H). In CP specimen from pancreatic resections, the acinar compartment showed robust DKK3 upregulation (Figure 3I). To conclude, DKK3 operates during acute pancreatic or hepatic damage, which might be relevant for injury‐induced repair of pancreas and liver in mice and men.

### 2.4 *Dkk3* Loss Attenuates Acute and Chronic Liver Injury

**Figure 4:**
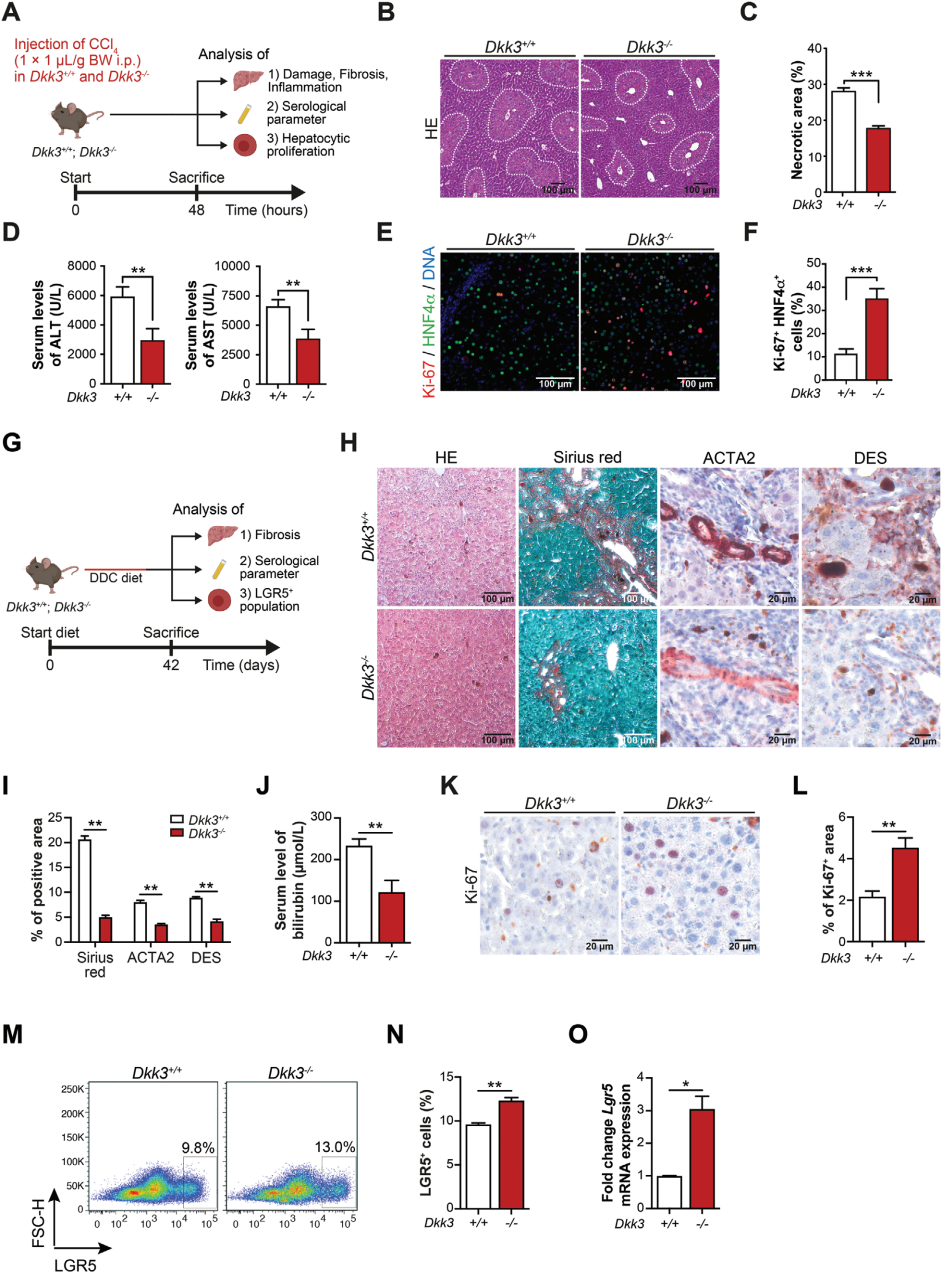
*Dkk3* deficiency inhibits CCl_4_‐induced acute and cholestasis‐induced chronic liver injury. A) Scheme illustrating the induction of acute liver injury. Wildtype (*Dkk3* ^+/+^) and *Dkk3*‐knockout (*Dkk3* ^−/−^) animals were treated with CCl_4_ and sacrificed during the regeneration phase 48 h after CCl_4_‐treatment. B) Representative pictures of liver HE‐staining with dotted line area indicating necrotic areas. C) Quantification of necrotic area in *Dkk3* ^+/+^ and *Dkk3* ^−/−^ animals (*n* = 7, bar graph shows mean + SEM, unpaired *t*‐test, *** *p* < 0.001). D) Analyses of liver‐specific enzymes ALT and AST in *Dkk3* ^+/+^ and *Dkk3* ^−/−^ 48 h after CCl_4_‐treatment. E) Representative pictures of Ki‐67 immunoreactivity in hepatocytes (HNF*α*). F) Quantification of Ki‐67^+^ hepatocytes per vision field in *Dkk3* ^+/+^ and *Dkk3* ^−/−^ animals (*n* = 5; bar graph shows mean + SEM, unpaired *t*‐test, ** *p* < 0.01). G) Schematic overview of the experiment analyzing the effect of *Dkk3* deficiency in DDC‐induced mouse models of liver fibrosis. H) Representative immunohistochemical images of HE, Sirius Red, ACTA2, and Desmin and Ki‐67 staining. I) Quantifications of Sirius Red, ACTA2, and Desmin staining in *Dkk3*‐knockout and control mice. J) Measurement of bilirubin content from *Dkk3*‐knockout and control mice. K) Representative immunohistochemical images of Ki‐67 staining. L) Quantification of Ki‐67‐positive cells in *Dkk3* knockout and control mice. M,N) Representative FACS analysis of LGR5^+^ LPCs in *Dkk3*‐knockout and control mice fed on DDC diet for 6 weeks. O) RT‐PCR analyses of *Lgr5* expression in *Dkk3*‐knockout and control murine livers. (Data are mean + SEM; two‐tailed Student's *t*‐test, * *p* ≤ 0.05 ** *p* < 0.01). DDC, 3,5‐diethoxycarbonyl‐1,4‐dihydrocollidine.

Numerous ways of liver regeneration such as i) hepatocyte self‐replication, ii) liver resident progenitor cells, or iii) progenitor‐like cells dedifferentiated from hepatocytes underpin the plasticity in liver regeneration, which is further triggered by the respective type of injury.^[^ ^32^, ^58^, ^59^ ^]^ To investigate this further, *Dkk3* ^+/+^ and *Dkk3* ^−/−^ mice received CCl_4_ treatment, a regimen predominantly causing hepatocytic damage to induce acute liver injury (**Figure** **4**A).^[^ ^33^, ^60^ ^]^ Interestingly, *Dkk3* ^−/−^ mice showed significantly less necrosis and reduced serum levels of liver enzymes (ALT, AST) while the amount of CD45^+^ leucocytes remained unchanged (Figure 4B–D and Figure S3A,B, Supporting Information). Compartment‐specific staining for Ki‐67 revealed that *Dkk3* ^−/−^ mice had significantly more Ki‐67^+^/HNF4*α* ^+^ double positive hepatocytes (Figure 4E,F), but not cholangiocytes (CK19^+^) (Figure S3C, Supporting Information).

Chronic DDC feeding resembles human cholestatic liver disease involving 1) biliary remodeling, which gives rise to progenitor, marks positive ductular reactions, 2) periductular fibrosis, and 3) chronic inflammation.^[^ ^61^ ^]^ This predominantly biliary damage results in the expansion of ductular reactions resembling induced LPC closely to the portal mesenchyme after three weeks and at later stages of fibrosis induction.^[^ ^35^, ^62^ ^]^ Accordingly, we induced cholestasis in wildtype and *Dkk3*‐knockout animals and quantified fibrosis and serological parameters (Figure 4G). Strikingly, *Dkk3* ^−/−^ mice displayed significantly reduced levels of fibrosis (Figure 4H,I and Figure S3D,E, Supporting Information), bilirubin serum levels (Figure 4J) and an overall increase in proliferation (Figure 4K,L), emphasizing decreased levels of liver damage in *Dkk3*‐knockout animals compared to the control mice. Moreover, we observed a significantly increased number of LGR5^+^ LPC in *Dkk3*‐null mice by flow cytometry and increased expression levels of Lgr5 by RT‐PCR (Figure 4M–O). Still, which population of liver cells is most susceptible for *Dkk3* ablation remains to be determined in more sophisticated, conditional liver injury models^[^ ^58^, ^62^ ^]^ as the stromal loss of *Dkk3* could also have impaired fibrosis in the DDC model.^[^ ^63^ ^]^

### 2.5 *Dkk3* Loss Increases Recovery Rate in AP

**Figure 5:**
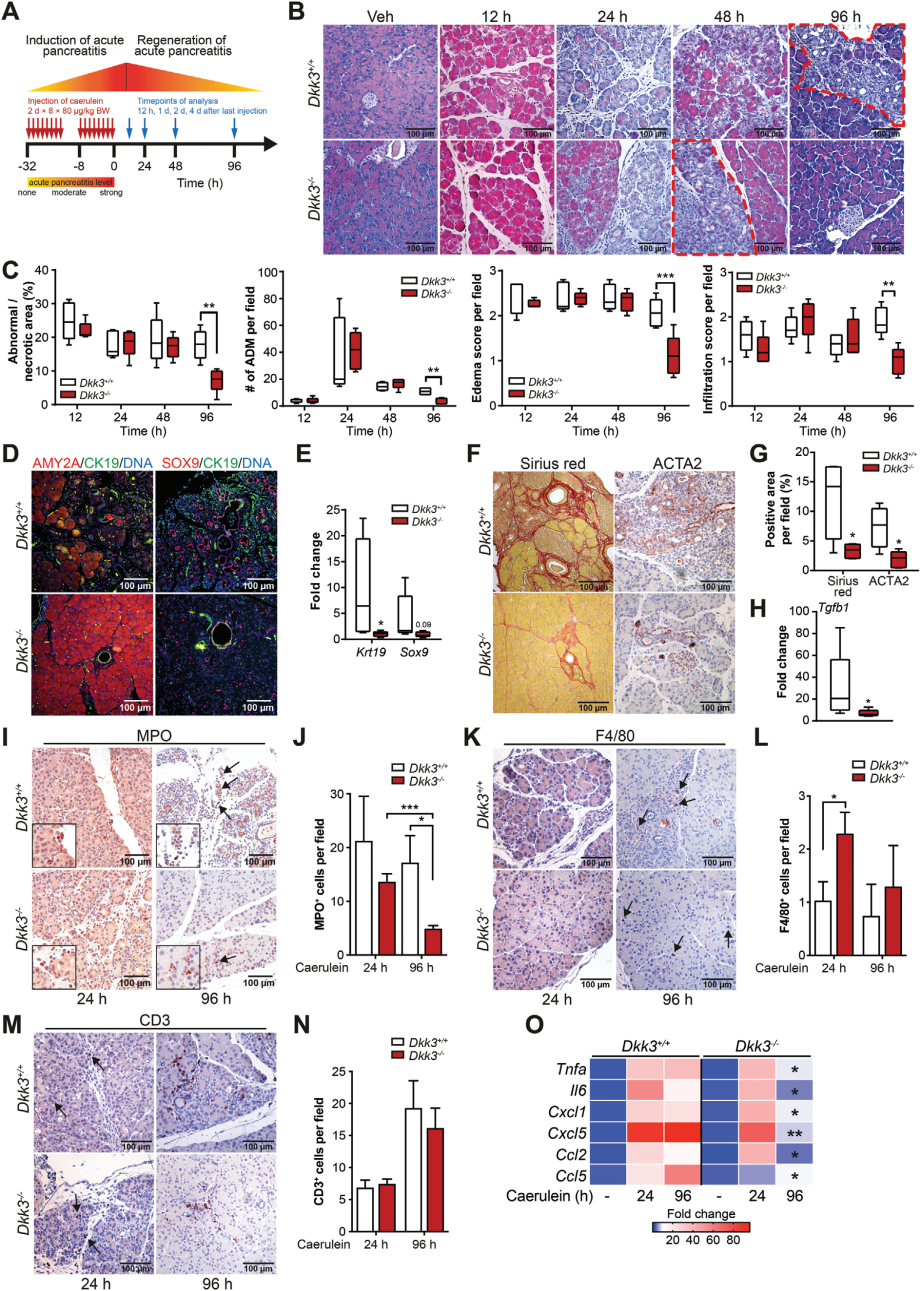
Loss of *Dkk3* accelerates regeneration of caerulein‐induced AP. A) Scheme illustrating the induction of AP. Wildtype (*Dkk3* ^+/+^) and *Dkk3*‐knockout (*Dkk3* ^−/−^) animals were treated with caerulein and sacrificed at the indicated time points. B) Representative pictures of HE‐staining of control (vehicle) and caerulein‐treated animals at different time points during regeneration of AP. Dotted area (red) indicates necrotic/abnormal area at indicated time points. C) Quantification of necrotic/abnormal area, ADM lesions, edema, and infiltration score 12, 24, 48, and 96 h after caerulein‐treatment (*n* ≥ 4). D) Representative pictures of IF staining of amylase (AMY2A), CK19, and SOX9 at day 4 upon caerulein‐treatment. E) RT‐PCR of ductal markers (*Krt19*, *Sox9*) at day 4 upon caerulein‐treatment. F) Representative pictures of histological staining of fibrotic content (Sirius red, ACTA2) at day 4 upon caerulein‐treatment. G) Quantification of Sirius Red and ACTA2^+^ area in caerulein treated *Dkk3* ^+/+^ and *Dkk3* ^−/−^ animals. H) RT‐PCR of fibrotic marker *Tgfb1* at day 4 upon caerulein‐treatment. I–N) Representative pictures of immunohistochemistry of I,J) MPO, K,L) F4/80, and M,N) CD3 specific antibodies, respectively at the indicated time points after caerulein‐treatment with corresponding quantifications. O) Heatmap illustrating RT‐PCR of inflammatory markers in control (vehicle) or caerulein‐treated (24 and 96 h) animals; (*n* ≥ 4 per group; box plots show median with min, max, bar graph means + SEM; statistics unpaired *t*‐test for histology (HE, Sirius red, IHC), Mann–Whitney U test for RT‐PCR, * *p* ≤ 0.05 ** *p* < 0.01, *** *p* < 0.001).

Next, we investigated a potential role of *Dkk3* during recovery from pancreatic injury mimicked by caerulein‐induced AP (**Figure** **5**A). AP is characterized by acinar cell death, de‐differentiation of acinar cells (ADM), inflammation, and fibrosis.^[^ ^21^ ^]^ After caerulein‐induced AP, *Dkk3* ^−/−^, and *Dkk3* ^+/+^ mice displayed similar levels of necrosis, ADM lesions, edema, and inflammatory infiltration scores (Figure 5B,C) at 12, 24, and 48 h post‐injury, suggesting that DKK3 expression does not influence the initial tissue damage. Interestingly, 96 h post‐intervention, *Dkk3* ^−/−^ mice showed significantly reduced necrosis, ADM lesions, edema, and infiltration scores compared to wildtype animals (Figure 5C), albeit serum levels of pancreas‐specific enzymes (lipase, amylase) were similar (Figure S4A, Supporting Information). These findings exhibit significantly reduced tissue damage already four days after injury, indicating enhanced regeneration in *Dkk3* ^−/−^ mice. At the same time point, the ductal markers *Krt19*/CK19 and *Sox9* (Figure 5D,E), which are usually elevated during ADM,^[^ ^21^ ^]^ were significantly decreased in *Dkk3* ^−/−^ animals. Of note, wildtype animals reached similar recovery status only after 7 days post‐induction (Figure S4B,C, Supporting Information). Significantly reduced damage was further substantiated by lower collagen content, alpha smooth muscle actin staining (ACTA2), and transforming growth factor β (*Tgfb1*) expression in *Dkk3*‐null animals 96 h after pancreatitis induction (Figure 5F–H), indicating less fibrosis at this time point. Fibrotic content at earlier time points was, however, similar in both genotypes (Figure S4D–G, Supporting Information). The amount of MPO^+^ neutrophils were also reduced 96 h post‐induction in *Dkk3* ^−/−^ versus *Dkk3* ^+/+^ mice (Figure 5I,J), with an opposing trend of F4/80^+^ macrophages. CD3^+^ T‐cells remained unchanged at both investigated time points (Figure 5K,N). Additionally, the expression levels of immunomodulatory cytokines, expressed by different cell types including injured acinar cells, activated fibroblasts, and immune cells (*Tnfa*, *Il6*, *Cxcl1*, *Cxcl5*, *Ccl2*, *Ccl5*) were similar 24 h but significantly reduced in *Dkk3* ^−/−^ mice after 96 h (Figure 5O). We conclude that the initial damage extent induced by caerulein treatment in *Dkk3*‐null pancreata is similar but regeneration is faster and more efficient.

### 2.6 Loss of *Dkk3* does not Employ TGF‐*β*‐ or ERK‐Signaling for Pancreatic Regeneration

**Figure 6:**
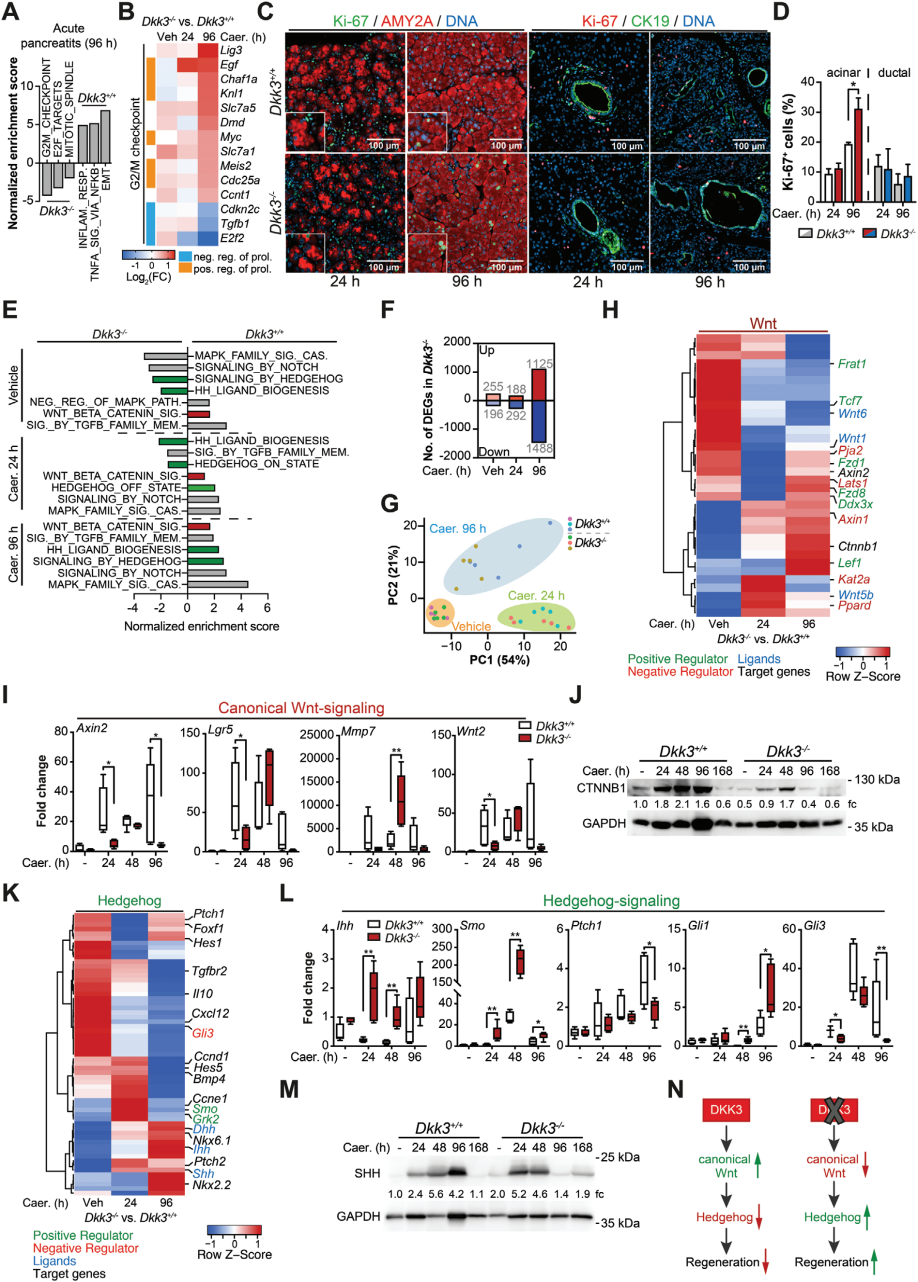
*Dkk3* loss restricts Wnt‐signaling activation and triggers Hh‐signaling. A) GSEA analysis of RNAseq data from wildtype (*Dkk3* ^+/+^) and *Dkk3*‐knockout (*Dkk3* ^−/−^) animals 4 days after induction of AP. Shown in the figure are three enriched Hallmark gene sets out of the top 10 most enriched gene sets in respective genotypes (*n* = 5). B) Heatmap illustrates DEG four days post‐caerulein treatment involved in proliferation (Hallmark_G2/M checkpoint). C) Representative pictures of Ki‐67/amylase (AMY2A) or Ki‐67/CK19 IF staining. D) Quantification of Ki‐67^+^ acinar (AMY2A^+^) and ductal (CK19^+^) cells in *Dkk3* ^+/+^ and *Dkk3* ^−/−^ animals, respectively (*n* = 5). E) GSEA analysis of RNAseq data from wildtype and *Dkk3*‐knockout animals for relevant signaling pathways responsible for regeneration. Hallmark and Reactome gene sets have been used for analysis. F) Bar graph shows DEGs (log2 fold change > 0.38 or ←0.38, *p*‐values < 0.05) in *Dkk3*‐knockout animals in comparison to wildtype. G) Principle component analysis during the regeneration of an AP of *Dkk3*‐proficient and *Dkk3*‐deficient animals. H) Heatmap shows differentially expressed genes of the Wnt‐signaling pathway in *Dkk3*‐null mice in comparison to wildtype. I) RT‐PCR analysis employing markers of canonical Wnt signaling pathway, and J) Western blot analysis of active beta‐catenin (CTNNB1) in control (vehicle) or caerulein‐treated mice at indicated time points. K) Heatmap shows differentially expressed genes of the Hh‐signaling pathway in *Dkk3*‐null mice in comparison to wildtype. L) RT‐PCR analysis employing markers of Hh signaling pathway, and M) Western blot analysis of SHH in control (vehicle) or caerulein‐treated mice at indicated time points. N) Scheme showing the effect of *Dkk3* on Wnt‐ and Hh‐signaling and its impact on regeneration of AP. (*n* = 3–6; bar graph means + SEM; box plot shows median with min, max, statistics unpaired *t*‐test for IF, Mann–Whitney U test for RT‐PCR, * *p* ≤ 0.05 ** *p* < 0.01).

To dissect the underlying mechanism of this improved regeneration, we performed RNA‐sequencing (RNA‐seq) analysis in wildtype and *Dkk3* knockout animals during the regeneration of AP. At 96 h after induction of pancreatitis, GSEA terms indicating proliferation (e.g., G2/M checkpoint) were enriched in *Dkk3*‐knockout animals, whereas wildtype animals still displayed an increased enrichment in fibrosis (e.g., EMT) and inflammation (e.g., Inflammatory response) (**Figure** **6**A). *Dkk3*‐null mice showed strong upregulation of genes positively regulating proliferation (e.g., *Egf*, *Myc*) and vice versa downregulation of negative regulators (e.g., *E2f2*) (Figure 6B). Time‐resolved acinar and ductal labeling with Ki‐67^+^ ascribed the increased proliferative response particularly to the acinar compartment and to the later time point when typically acini undergo redifferentiation^[^ ^21^ ^]^ (Figure 6C,D). A plethora of developmental pathways such as Wnt‐, Notch‐, and Hh‐signaling can promote acinar re‐differentiation and proliferation.^[^ ^21^ ^]^ Besides Wnt‐signaling, DKK3 has been shown to regulate other pathways such as TGF‐*β*‐ and ERK‐signaling.^[^ ^38^, ^45^, ^64^ ^]^ To screen potentially involved pathways, we performed gene set enrichment analysis (GSEA) at respective time points (vehicle, 24 and 96 h after induction of pancreatitis) and found enrichment for TGF‐*β*‐, Notch‐ and MAPK‐signaling changing with time point and genotype (Figure 6E). However, validation by immunoblotting for the key effectors such as p‐SMAD2 and p‐ERK1/2 did not reveal any conclusive pattern (Figure S5A–C, Supporting Information). Of note, downregulation of the TGF‐*β* downstream target Pai1 at 96 h likely mirrors decreased fibrosis in *Dkk3*‐null animals (Figure S5D, Supporting Information). We conclude that, although slightly regulated, neither TGF‐*β*‐ nor ERK‐ signaling seem to operate as signaling modules during improved pancreatic regeneration upon *Dkk3* loss.

### 2.7 *Dkk3* Loss Limits Canonical Wnt‐Signaling During Pancreatic Regeneration

As GSEA enrichment for Wnt‐ and Hh‐signaling, both being well established during pancreatic regeneration,^[^ ^20^, ^21^, ^65^ ^]^ changed across individual time points and genotypes, we further dissected their contribution to improved repair in *Dkk3*‐null mice (Figure 6E). Wnt‐signaling was described to regulate rather cell growth^[^ ^21^ ^]^ while active Hh‐signaling diminishes fibrosis, promotes proliferation, and is necessary for proper acinar re‐differentiation.^[^ ^20^, ^65^ ^]^ The involvement of DKK3 in modulation of Wnt‐signaling has been extensively described.^[^ ^27^, ^28^, ^66^ ^]^ As simple GSEA is frequently hampered in discriminating the repression or activation of a given pathway, we determined differentially regulated genes (DEG) at individual time points.^[^ ^67^ ^]^ In line, the amount of DEGs increased over time with highest numbers at 96 h after induction of pancreatitis (Figure 6F), an observation in concordance with our morphological observations. In addition, separation of wild type and *Dkk3*‐null transcriptomes according to their principal components increased over time (Figure 6G). We specifically focused on the regulatory patterns of Wnt and Hh genes and found relevant key players of these pathways differentially regulated (Figure 6H,K). In particular, negative mediators of Wnt‐signaling (e. g., *Axin1*, *Kat2a*, *Ppard*) were upregulated, whereas positive mediator (e.g., *Lef1*, *Tcf7*, *Fzd1*) and Wnt‐ligands (e.g., *Wnt1*, *Wnt6*) were negatively impacted (Figure 6H).

The downregulation of activator of the Wnt‐/*β*‐catenin pathway alongside with the upregulation of Wnt inhibitors in *Dkk3*‐null mice during the recovery phase indicate substantially attenuated canonical Wnt‐signaling. Further validation of other (canonical) Wnt target genes (*Axin2*, *Lgr5*, *Mmp7*, *Wnt2*) by RT‐PCR and active *β*‐catenin (CTNNB1) by Western blot confirmed this observation but also illustrated residual but attenuated activation in *Dkk3*‐null pancreata (Figure 6I,J). Non‐canonical Wnt‐signaling target genes (*Cdc42*, *Vangl2*) remained at least at 24 h unchanged (Figure S5E, Supporting Information). Residual canonical Wnt activation might happen due to crosstalk of other signaling pathways, for example, Hh.^[^ ^68^ ^]^ Overall, these data indicate that *Dkk3* is required for injury‐induced Wnt‐signaling activation. Since no differences were observed in non‐canonical Wnt‐signaling target genes (*Cdc42*, *Vangl2*) at 24 h (Figure S5E, Supporting Information), our data suggest that the regulatory effect of *Dkk3* is limited to canonical Wnt‐signaling during pancreatic regeneration.

### 2.8 Increased Hh‐Signaling follows *Dkk3* Loss to Foster Pancreatic Regeneration

Although a direct link between DKK3 and Hh‐signaling has not yet been reported, our differential GSEA patterns of Hh terms prompted us to specifically assess Hh genes (Figure 6E).

Particularly, Hh‐ligands (e.g., *Shh*, *Ihh*, *Dhh*) and positive regulators (e.g., *Smo*, *Grk2*) were upregulated, following an increase of the expression of the Hh‐target genes (e.g., *Ccnd1*, *Ccne1*, *Nkx2.2*, *Nkx6.1*) (Figure 6K), strongly suggesting a positive regulation of Hh‐signaling in *Dkk3*‐null mice during the regeneration phase.

Validation via RT‐PCR and/or western blotting confirmed strong upregulation of the Hh‐ligands Indian hedgehog (*Ihh*) and Sonic hedgehog (SHH) as well as the Hh‐receptor Smoothened (*Smo*) in *Dkk3* ^−/−^ animals already 24 h after pancreatitis induction, followed by a significant increase of the target gene glioma‐associated oncogene 1 (*Gli1*) 96 h post‐injury (Figure 6L,M and Figure S5F, Supporting Information). Intriguingly, the low expression of the Hh‐target gene Patched1 (*Ptch1*), a known negative regulator, was only slightly upregulated in *Dkk3* ^−/−^ mice, but significantly lower than compared to wildtype animals 96 h post‐injury (Figure 6L).

Wnt‐signaling has been shown to regulate the transcriptional expression of the GLI3 repressor protein.^[^ ^69^ ^]^ *Gli3* mRNA levels were significantly decreased in *Dkk3* ^−/−^ animals compared to *Dkk3* ^+/+^ mice after caerulein treatment and only increased 48 h post‐induction at the time of augmented Wnt activity (Figure 6L). Thus, our data suggest that upon induction of AP, *Dkk3* deletion restricts Wnt activation and subsequently the transcription of the Hh‐repressor gene *Gli3*. This, in turn, leads to an earlier activation of Hh‐signaling and thus an increased recovery rate (Figure 6N).

**Figure 7:**
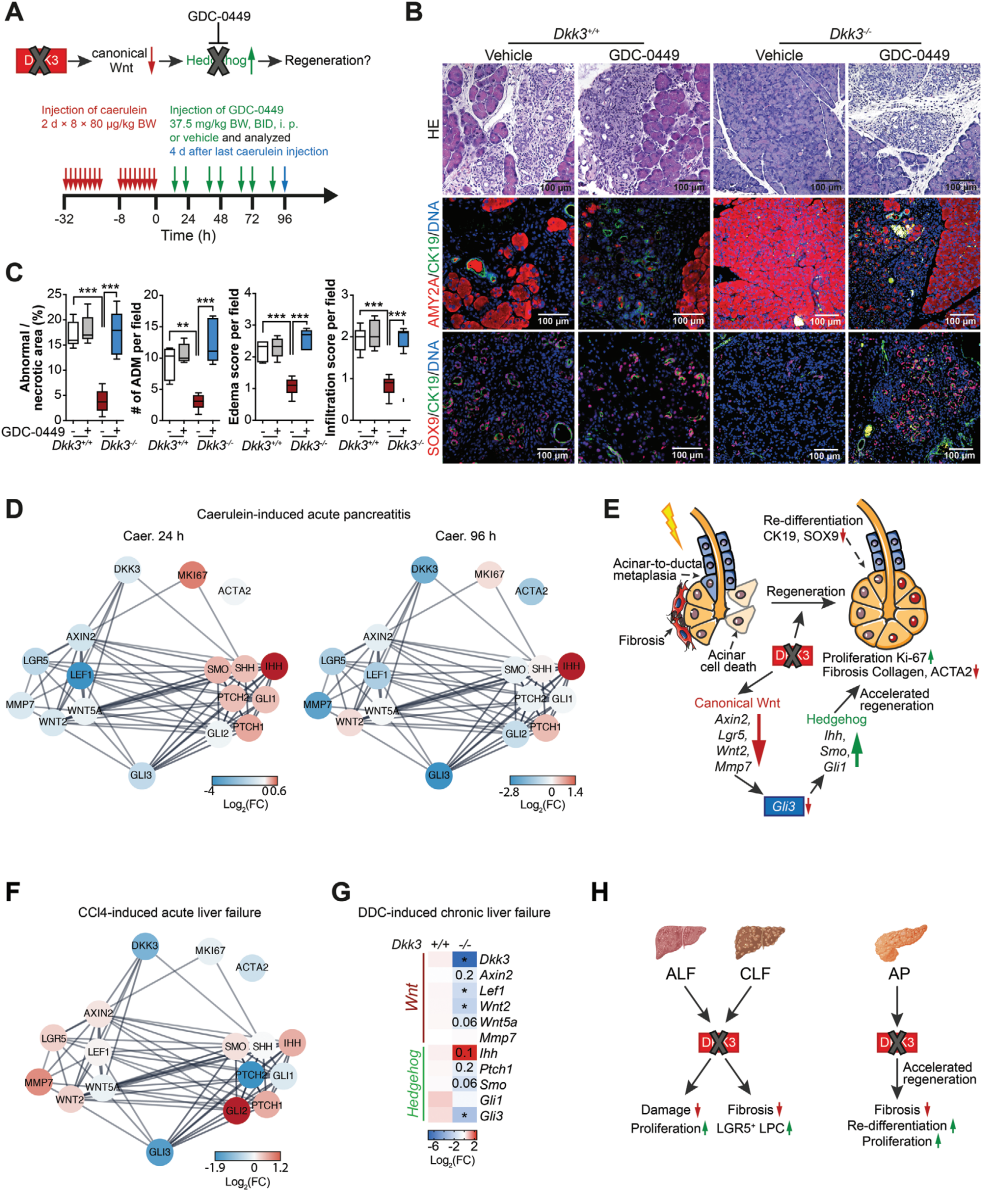
Hh inhibition worsens regeneration of AP in *Dkk3* ^−/−^ mice. A) Scheme depicting the procedure of depletion of Hh‐signaling in *Dkk3* ^+/+^ and *Dkk3* ^−/−^ animals. Animals were treated with caerulein, followed by GDC‐0449 treatment during regeneration phase, and sacrificed after four days from last caerulein‐treatment. B) Representative pictures of HE, amylase, CK19, and SOX9 IF staining in control or GDC‐0449 treated animals. C) Quantification of necrotic area, ADM lesions, edema, and infiltration score of control or GDC‐0449 treated mice (*n* = 5; box plot shows median with min, max, unpaired *t*‐test, ** *p* < 0.01, *** *p* < 0.001). D) STRING network shows the connection between DKK3, Wnt‐, Hh signaling, and proliferation. Node color indicates the changes expression level (log2 fold) from RNAseq of *Dkk3*‐knockout animals in comparison to wildtype in mice with AP at respective time points. E) Graphical illustration of the impact of *Dkk3* loss on the regeneration of caerulein‐induced AP. F) STRING network illustrates the connection between DKK3, Wnt‐, Hh‐signaling, and proliferation as in D from mice with CCl_4_‐induced ALF. G) RT‐PCR analysis of canonical Wnt‐ and Hh‐signaling pathway in *Dkk3*‐knockout and control murine livers fed with DDC for 6 weeks (*n* = 4; Mann–Whitney U test, * *p* ≤ 0.05 ** *p* < 0.01). H) Scheme illustrates the impact of the loss of *Dkk3* on liver and pancreatic injury.

To further challenge the *Dkk3* loss‐dependent activation of Hh‐signaling, we treated wildtype and *Dkk3* ^−/−^ mice with the Smo‐Hh‐inhibitor GDC‐0449 (Vismodegib) during the regeneration of caerulein‐induced AP (**Figure** **7**A). As expected, GDC‐0449 significantly increased necrosis, edema, and inflammatory infiltration 96 h after caerulein treatment only in *Dkk3* knockout animals, whereas the extent of damage in wildtype mice remained nearly unaffected at this time point (Figure 7B,C). Furthermore, GDC‐0449 treatment decreased expression of the acinar cell marker amylase and increased expression of the ductal markers CK19 and SOX9 in *Dkk3*‐null animals (Figure 7B), reversing the pro‐regenerative effects of *Dkk3* loss. This suggests that organ regeneration after AP is potentiated by *Dkk3* loss, which in turn releases an Hh‐signaling roadblock to boost pancreatic acinar redifferentiation and thus regeneration.

### 2.9 Shared Pathways During Pancreatic and Liver Regeneration in the Absence of DKK3

Our results indicate that loss of *Dkk3* becomes particularly relevant during tissue regeneration after injury but does not seem to affect normal tissue homeostasis. To mechanistically translate these data from the pancreas to the liver, both developing from the same common progenitor,^[^ ^70^ ^]^ we employed the STRING database to generate networks showing potential connections and expression changes across DKK3, Wnt, and Hh genes in *Dkk3*‐knockout animals upon pancreatic and hepatic injury (Figure 7D). In the pancreas, the constructed networks support our experimental observations that *Dkk3* loss triggers a complex interplay between Wnt‐ and Hh‐signaling indicating that restricted canonical Wnt‐activation triggers Hh activation in particular 24 h after injury‐induction (Figure 7D). This translates into faster tissue regeneration via increased proliferation of the acinar compartment, improved acinar re‐differentiation and reduced tissue fibrosis (Figures 5, 6, and 7E).

We also performed RNA‐seq analysis from CCl_4_‐treated livers and constructed a similar, but not time‐resolved, STRING network (Figure 7F). Again, the Hh repressor *Gli3* was downregulated alongside with up‐regulation of the Hh‐ligand *Ihh* in *Dkk3*‐knockout animals (Figure 7F). RT‐PCR analysis in the DDC revealed a similar but not entirely concordant regulation pattern (Figure 7G). Although, STRING networks of pancreas and liver showed a certain degree of overlap, changes in Wnt‐ and Hh‐signaling genes triggered upon *Dkk3* loss appear to differ in time and intensity between the pancreas and the liver. Additional time‐resolved RNA‐seq analysis in both DDC and CCl_4_‐treated mice will be necessary to unravel this potential interplay more precisely.

In summary, our data identify *Dkk3* as a roadblock in liver and pancreas repair/regeneration upon injury. Specifically, *Dkk3* ablation limits fibrosis and liver damage mediated by accelerated hepatocyte proliferation and/or LPC expansion depending on the liver insult (Figure 7H). Similarly, *Dkk3*‐null pancreata regenerate faster and with attenuated fibrotic reaction via improved acinar redifferentiation and proliferation (Figure 7H). Quantification of DKK3 levels in blood and tissue from patients suggests similar mechanism in humans.

## 3 Discussion

Here we reveal *Dkk3* as a relevant roadblock during somatic cell reprogramming and gastrointestinal tissue repair by integrating a customized shRNA screen on a three‐factor based (OCT3/4, KLF4, SOX2) reprogramming platform followed by a hypothesis‐driven validation in distinct organs. The hypothesis builds on shared regulatory elements between reprogramming and tissue repair or regeneration.^[^ ^71^ ^]^ The bioinformatics approach considering antithetic ranks of a given shRNA in non‐reprogrammed and reprogrammed cells allowed the reduction of false‐positive hits and therefore the direct validation of data using *Dkk3* ^−/−^ mice and matching primary cell cultures. Indeed, loss of *Dkk3*, a top candidate gene in our screen markedly enhanced iPSC formation. In line with the notion that genes acting as “roadblocks” during reprogramming do not necessarily have to be relevant to maintain pluripotency, de novo derivation of ESCs from blastocysts occurred similarly robustly in *Dkk3* ^+/+^ and *Dkk3* ^−/−^ cells. Finally, our screen identified a number of other candidate “roadblock genes” (e.g., a subset of *Hox* genes), which will be a relevant repository to study further genes involved in both reprogramming and tissue repair/regeneration.

While DKK3 is known to regulate Wnt‐ and TGF‐*β*‐signaling, two crucial pathways in tissue repair and homeostasis,^[^ ^72^, ^73^, ^74^ ^]^ no specific role for DKK3 in tissue regeneration has been described so far. Our study reveals that *Dkk3* loss results in increased numbers of iPSCs during somatic reprogramming of MEFs without affecting pluripotency levels. This could be attributed to the fact that DKK3 is expressed and secreted in fibroblasts^[^ ^63^ ^]^ and cancer‐associated fibroblasts^[^ ^75^, ^76^ ^]^ but not in embryonic stem cells.^[^ ^45^ ^]^ DKK3, therefore, might act as a paracrine barrier toward a pluripotent state. Furthermore, we could not observe differences in spontaneous differentiation into the three germ layers in *Dkk3* ^−/−^ animals. However, we did not focus on complete lineage commitment,^[^ ^77^ ^]^ as DKK3 was shown to be important for ESC differentiation into smooth muscle cells in response to defined stimuli.^[^ ^45^ ^]^

We primarily aimed to implement the reprogramming process as a resource tool to identify new regulators of tissue repair. After delineating the expression of DKK3 in the liver and pancreas, the impact of *Dkk3* deletion was characterized in colony formation assays and organoid cultures of the respective tissue entities. Intriguingly, no relevant genotype‐related differences in pancreas or liver could be detected.

Conversely, our study reveals for the first time that *Dkk3* loss is associated with better tissue regeneration after hepatic and pancreatic injury. Still similar mechanisms can be expected from intestinal injury, since DKK3 was also upregulated in the blood of humans suffering chronic inflammatory bowel disease as well as in murine experimental colitis models (not shown). Intriguingly, the upregulation of DKK3 during injury in pancreas and liver already suggests a possible role in preventing an overshoot of pathways during regeneration. In our model, regeneration in *Dkk3* ^−/−^ mice is associated with impaired Wnt‐signaling activity. This is in line with recent studies demonstrating that DKK3 potentiates cell type‐specific Wnt‐signaling through interaction with Kremen receptors.^[^ ^27^, ^78^ ^]^ Interestingly, Wnt pathway activity was shown to directly regulate the transcription of *Gli3*, a repressor of Hh‐signaling.^[^ ^69^ ^]^ In line with this, we found reduced expression levels of *Gli3* in *Dkk3* ^−/−^ animals, most likely because of the restricted Wnt activation. This translated into earlier Hh pathway activation in *Dkk3* ^−/−^ mice during pancreatic regeneration. Elevated Ihh levels and the activation of Hh‐signaling in hepatic stellate cells also accelerated liver regeneration upon partial hepatectomy^[^ ^18^ ^]^ and other modes of liver injury.^[^ ^79^, ^80^ ^]^ Of note, increased bipotent colony formation of *Dkk3* ^−/−^ fetal liver progenitors observed in our work is along the lines of previous data suggesting a role of DKK3 in hepatic differentiation.^[^ ^55^ ^]^ Moreover, increased Hh‐signaling as suggested by our STRING networks might control viability of LPCs^[^ ^81^ ^]^ and facilitates YAP1 expression, which, in turn, promotes the hepatic progenitor cell compartment.^[^ ^18^, ^82^, ^83^ ^]^ Of note, YAP signaling might be a driver of the increased ductular reaction alongside with LGR5‐positive progenitor expansion in our DDC model. However, the ultimate repair mechanism during liver injury is still a matter of debate discussing hepatocyte‐self replication versus de‐differentiation versus various types of tissue resident stem cells and our data might deserve investigation of this potential mechanism in future studies.^[^ ^51^, ^52^, ^58^ ^]^

Our findings also extend the previous studies on Hh‐signaling and its role during acinar regeneration. Hh‐signaling has been shown to drive acinar cell proliferation, but also reverses ADM to restore cell homeostasis entirely,^[^ ^20^, ^65^ ^]^ which can also be mediated (at least partially) by increased numbers of progenitor‐like cells in *Dkk3*‐null animals. Single‐cell transcriptome analysis uncovered cell plasticity within acinar cells including the presence of progenitor‐like cells favoring acinar cell regeneration.^[^ ^49^, ^84^ ^]^ However, factors limiting regeneration might also be relevant in the prevention of dysplastic growth and subsequent cancer formation.^[^ ^85^ ^]^ Indeed, our screening library has been specifically designed to particularly address the potential connection of the target genes to cancer. DKK3 emerged as putative tumor suppressor, as suggested by other studies before.^[^ ^86^, ^87^ ^]^ In contrast, the suppression of stromal DKK3, either genetically‐ or antibody‐mediated, inhibits progression of *Trp53* ^−/−^ pancreatic cancers and prolongs survival.^[^ ^75^ ^]^ On the other hand, DKK3 overexpression sensitizes toward gemcitabine treatment.^[^ ^88^ ^]^ Thus, DKK3 may act either as a friend or as a foe, depending on the target tissue. Thus, targeting DKK3 might be an interesting therapeutic approach not only for pancreatic cancer, but also for CP. Other tumor suppressors such as p21 have been shown to act in a similar manner.^[^ ^89^, ^90^ ^]^

Nevertheless, our translational efforts in human specimen impel future studies in more translational human *DKK3* loss‐of‐function models taking into account potential side effects in terms of dysplastic triggers to stratify DKK3 as a potential target during gastrointestinal injury.

## 4 Conclusion

In conclusion, our current study reveals an essential role of *Dkk3* during the regeneration after acute liver injury and pancreatitis, and establishes a new link between DKK3, canonical Wnt‐signaling and Hh‐signaling. Using iPSC reprogramming, organoid assays and genetically engineered mouse models, we demonstrate that i) *Dkk3* deletion reduces liver damage and improves tissue recovery in AP, particularly in the acinar compartment; ii) the improved tissue regeneration in *Dkk3* ^−/−^ mice is regulated via augmented Hh activity, potentiating the regenerative response; and iii) *Dkk3* may thus enhance the activation of canonical Wnt‐signaling beyond the physiological levels. Overall, we propose DKK3 as a key regulator during gastrointestinal tissue regeneration and repair, and its modulation might be worth implementing in a clinical setting in the future.

## 5 Experimental Section

### Ethics Statement and Study Design

In animal studies, mice were randomly assigned to control and treatment groups. The number of animals are specified in each figure legend. *Dkk3* knockout mice (*Dkk3* ^tm1Cni^) were obtained from Christof Niehrs^[^ ^29^ ^]^ and kept in a complex B6;129×1‐*Dkk3* ^tm1Cni^ background by crossings with C57BL/6J. Tail‐derived DNA and specific primers^[^ ^29^ ^]^ were used for mouse genotyping. *Dkk3* wildtype littermates were used as controls. All animal work was conducted either under ethical and animal protection regulations of the German animal protection law and were previously approved by the respective governmental review board of the state of Baden‐Württemberg (TVA‐1461, O.195‐5). DDC mouse model was conducted according to the guidelines of the Institutional Animal Care and Use Committee of the Hannover Medical School, Germany. The human patient material used in this study was either provided by the biobank of the University Hospital of Ulm following the regulations of the Biobank and the vote of the Ethics Committee of the University of Ulm (Ethics no. 159/19) or by the University Medicine Greifswald (Ethics no. III UV 91/03b). Patient's characteristics, which were selected in this study, are included in Table S1, Supporting Information. Healthy subject group was used as control.

### TSG Screen

MEFs were infected with shRNA from a library^[^ ^37^ ^]^ of 700 tumor suppressor genes (TSG) harboring a GFP reporter, followed by reprogramming into iPSCs with concentrated polycistronic OKSM (OCT3/4, KLF4, SOX2, c‐MYC, four factor‐based reprogramming) lentivirus. Infection rate and cell number were adjusted to avoid double integration. At day 13, the reprogramming cells were stained for SSEA1 and sorted for only GFP^+^ cells and GFP^+^ and SSEA1^+^ double positive cells. Genomic DNA from both populations was isolated using standard techniques, subsequently sent for deep sequencing and analyzed for abundant shRNAs. Bioinformatics analysis was done using R software. For candidate selection, read counts were ranked and ranks were compared between iPSC cell (repro) and non‐iPSC cells (non‐repro). Genes ranked within the top 20% in the non‐repro list and the lower 50% in the repro list were considered enriched. For heatmap display read counts were normalized and log2 transformed.

### Generation of iPSCs

Reprogramming of MEFs into iPSCs was performed as followed: One day prior infection, MEFs were seeded on a 0.2% gelatin‐coated plate (4 × 10^4^ cells/12‐well or 1×10^6^ cells/6‐well). Next day, MEFs were transduced with concentrated polycistronic OKS (OCT3/4, KLF4, SOX2) lentivirus harboring a Td‐tomato20 (11.9×10^7^ proviral hOKS copies/µL), 4 µL per well of 12‐well plate or 8 µL per well of 6‐well plate, together with 8 µg mL^−1^ polybrene (Sigma‐Aldrich) in 0.5 mL and 1 mL ES‐Feeder Medium, respectively. After 8 h of incubation at 37 °C, ES‐feeder medium was refreshed and changed daily. The medium was changed to KOSR‐ES‐feeder medium at day 6 and cells were either analyzed by flow cytometry or stained for alkaline phosphatase expression at day 13 of reprogramming.

### In Vitro Colony‐Formation Assay of LPCs

Detailed procedure of isolation of fetal and adult LPC is described in the Supporting Information. After sorting of LPCs (adult or fetal), single cell LPCs were cultivated in collagen type‐I‐coated 96‐well plates in standard medium containing conditioned medium derived from E14.5 fetal liver cells and DMEM/F12 (ratio 1:1, Gibco Life Technologies) supplemented with 10% FBS, 2 mm l‐glutamine (Gibco Life Technologies), 10 mm nicotinamide (Sigma‐Aldrich), 10^−7^ m dexamethasone (Sigma‐Aldrich), 2.5 mm HEPES (Gibco Life Technologies), 1× Insulin‐Transferrin‐Selenium (Gibco Life Technologies), 1× NEAA (Gibco Life Technologies), 1× P/S (Sigma‐Aldrich), as well as 40 ng mL^−1^ hepatocyte growth factor (PeproTech), 20 ng mL^−1^ epidermal growth factor (PeproTech), and 20 µm Y‐27632 (Rho‐associated kinase inhibitor; Ascent Scientific). After three weeks of cultivation, colony number (> 50 cells) was determined. Nature of colonies were characterized morphologically as well as by IF staining for CK19 (cholangiocyte) and Albumin (hepatocyte).

### Isolation and Cultivation of PDO

The pancreas from *Dkk3* knockout or wildtype mice was minced by subsequent digestion with collagenase/dispase (Roche) for 30 min at 37 °C followed by incubation in accutase (Sigma‐Aldrich) for 30 min at 37 °C. Afterward, cells were filtered through a 40 µm cell strainer (Greiner bio‐one). After centrifugation, cells were resuspended in pancreatic ductal organoid cell (PDC) medium supplemented with 5% growth factor reduced (GFR) Matrigel (Corning, 354 230) and plated onto GFR‐Matrigel coated 12‐well plates. The PDC medium is based on the protocol from Reichert et al.^[^ ^91^ ^]^

### Acute and Chronic Liver Damage

To induce ALF, mice (8–12 weeks) were treated intraperitoneal with a single dose of CCl_4_ at a concentration of 1.0 µL CCl_4_ / g bodyweight of mice diluted 1:3 in ClinOleic (Baxter Germany GmbH). Acute liver damage was assessed 48 h after CCl_4_‐injection by histopathological quantification of necrosis (HE), immune cell infiltration (CD45‐IHC), and proliferation index (Ki‐67) in hepatocytes (HNF4*α*) and cholangiocytes (CK19).

To establish cholestasis‐induced liver fibrosis in vivo, mice were fed on 0.1% DDC diet for 6 weeks. The level of fibrosis was assessed by IHC and RT‐PCR. LPC recruitment was determined by FACS and RT‐PCR.

### Isolation and Flow Cytometry of LGR5^+^ LPCs

Isolation and determination of LGR5 positive LPCs were performed as previously described.^[^ ^92^ ^]^ Liver was perfused by a two‐step collagenase (Roche) perfusion method. After passing the digested liver, which was resuspended in 50 mL DMEM, through a 100 µm nylon mesh, the cell suspension was centrifuged at 50 g for 5 min at 4 °C. The supernatant containing non‐parenchymal liver cells was collected and centrifuged at again at 300 g for 5 min at 4 °C. Murine non‐parenchymal liver cells were probed with anti‐LGR5 antibody (Abcam, ab75732) and subsequently labeled by Alexa‐594 conjugated goat anti‐rabbit secondary antibody. LPR5^+^ cells were analyzed using MoFlo XDP machine.

### AP and CP

AP in mice (8–12 weeks) was induced by hourly injection (eight times) of 80 µg caerulein/kg bodyweight (Sigma‐Aldrich) dissolved in PBS or vehicle (PBS only) on two consecutive days. Mice were sacrificed within the regeneration phase after 12 h, 1 day, 2 days, and 4 days after last injection with caerulein. Mice treated with vehicle were sacrificed one day post‐injection. To ablate Hh‐signaling, *Dkk3* ^−/−^ mice were treated intraperitoneally with 37.5 mg/kg bodyweight of Hh inhibitor GDC‐0449 (Selleckchem) or vehicle (DMSO) twice a day (37.5 mg/kg bodyweight, BID 10 h, i.p.) 16 h after caerulein‐treatment. Pancreas was removed and pancreatic damage was assessed by histology, immunohistochemistry, and RT‐PCR analysis. Pancreas tissue specimen with CP was kindly provided by the laboratory of Prof. Wirth (University of Ulm). Induction was previously described.^[^ ^93^ ^]^

### Histology, Immunohistochemistry, Immunofluorescence

Histology, immunohistochemistry, and IF were performed using standard protocols. A more detailed description including antibodies (Table S2, Supporting Information) used in this study is available in the Supporting Information.

### RNA Extraction, cDNA Synthesis, and RT‐PCR

RNA extraction, cDNA synthesis for RT‐PCR, and RT‐PCR were conducted using standard methods. A more detailed description including primers (Table S3, Supporting Information) used in this study is available in the Supporting Information.

### Histopathological Analysis and Scoring of AP and Liver Injury

Caerulein‐induced AP was classified according to morphological histopathological criteria.^[^ ^94^ ^]^ In line with these criteria, edema and inflammatory infiltration per visual field were scored from zero to three (none 0, mild 1, moderate 2, severe 3), as well as necrotic area and number of ADM per visual field from at least 10 images at high‐power magnification were quantified. Fibrotic area was quantified by determination of Picrosirius Red and ACTA2 positive area in IHC‐stained tissue sections. Proliferation was assessed by determining Ki‐67‐proliferation index in respective tissue compartments (acinar vs ductal, hepatocytes vs cholangiocytes). Necrotic area and inflammatory infiltration of liver was determined by quantification of necrotic area from HE or CD45‐positive area from IHC per visual field.

### Enzyme‐Linked Immunosorbent Assay

Serum levels of secreted DKK3 were determined using DKK3 Human ELISA Kit (Thermo Fisher Scientific) according manufacturer guidelines. Absorbance was measured at Tecan Infinite M200 Pro.

### RNAseq Analysis

The initial library preparation for bulk‐sequencing of poly(A)‐RNA was performed as described previously.^[^ ^95^ ^]^ Briefly, the barcoded cDNA of each sample was generated using Maxima RT polymerase (Thermo Fisher Scientific) and oligo‐dT primer containing barcodes, unique molecular identifiers (UMIs) as well as an adaptor. cDNA ends were extended using a template switch oligo (TSO), following full‐length cDNA amplification using primers that bind to the TSO‐site and the adaptor. Further cDNA fragmentation was performed using NEB UltraII FS kit. After end‐repair and A‐tailing a TruSeq adapter was ligated and 3’‐end‐fragments were finally amplified with Illumina using the P5 and P7 overhang primers. In comparison to Parekh et al. (2016),^[^ ^95^ ^]^ the P5 and P7 sites were exchanged to allow sequencing of the cDNA in read1 and barcodes and UMIs in read2 to achieve a better cluster recognition. The library was sequenced on a NextSeq 500 (Illumina) with 67 cycles for the cDNA in read1 and 16 cycles for the barcodes and UMIs in read2. Data was processed using the published Drop‐seq pipeline (v1.0) to generate sample‐ and gene‐wise UMI tables.^[^ ^96^ ^]^ Reference genome (GRCm38) was used for alignment. Transcript and gene definitions were used according to the GENCODE Version M25.

### STRING Network

Protein‐protein interaction network was generated using STRING database and Cytoscape software version 3.6.0. The minimum required interaction score was set on default (medium confidence 0.400). Log2 fold change, calculated from RNAseq data, of *Dkk3*‐knockout animals represents the node color.

### Gene Set Enrichment Analysis

GSEA was performed using the Reactome and hallmark data sets from the Molecular signatures database v7.0 (MSigDB, Broad Institute; http://software.broadinstitute.org/gsea/msigdb). Significant enrichments were defined false discovery rate < 0.25.

### Statistical Analysis

If not otherwise stated in the figure legends, two‐tailed unpaired *t*‐test was performed for quantification of histological analysis and ELISA and Mann‐Whitney U test was used for RT‐PCR. Significance has been addressed as followed: * *p* ≤ 0.05, ** *p* < 0.01, *** *p* < 0.001 from at least three biologically independent experiments. The data are presented as the means + SEM or as box blots with min and max values as stated in the figure legends. The sample size (*n*) for animal models were statistically determined based on previously performed similar experiments. For each experiment, the exact sample size is indicated in the figure legend. Data analyses were conducted using GraphPad Prism Version 5 or version 8. Data analyses for RNA‐seq was performed using R software. Heatmaps were partially generated using the “Heatmapper” web tool.^[^ ^97^ ^]^ Rows were clustered using average linkage with Pearson distance measurement. Additionally, rows were normalized using row scale.

## Conflict of Interest

The authors declare no conflict of interest.

## Author Contributions

P.C.H. and A.K. jointly supervised this work. F.A., P.C.H., S.L., and A.K. conceived the study, designed the experiments, analyzed the data, and wrote the manuscript. F.A., X.Z., D.S., J.G., W.B., S.F.K., T.S., N.D., P.U.M., R.R., and U.T. performed the experiments and/or analyzed data. E.R. and L.P. performed histopathological analysis. R.O., Q.L., R.R., J.M.K., and H.A.K. performed RNAseq and/or conducted bioinformatical analysis. A.L., N.A., and S.L. provided technical support. F.U.W., M.M.L., E.Z., and A.K.B. provided, collected, and analyzed clinical patient parameters. A.I., A.L., M.O., A.D.S., and T.S. contributed to study design. All authors contributed to the review and editing of the manuscript

## Supporting information

Supporting Information

## Data Availability

Research data are not shared.
