# Supplementary material for: Functional Genomic Screening During Somatic Cell Reprogramming Identifies DKK3 as a Roadblock of Organ Regeneration
Source: Adv Sci (Weinh). 2021 May 13;8(14):2100626. doi: 10.1002/advs.202100626 (PMC8292873; doi:10.1002/advs.202100626)
Supplement: Supplementary file 1 — Supporting Information [file ADVS-8-2100626-s001.pdf]

© 2021 Wiley-VCH GmbH

ADVANCED SCIENCE
Open Access

Supporting Information

for *Adv. Sci.*, DOI: 10.1002/advs.202100626

Functional Genomic Screening During Somatic Cell Reprogramming Identifies DKK3 as a Roadblock of Organ Regeneration

Frank Arnold[1], Pallavi U Mahaddalkar[2], Johann M. Kraus[3], Xiaowei Zhong[4], Wendy Bergmann[5], Dharini Srinivasan[1], Johann Gout[1], Elodie Roger[1], Alica K. Beutel[1], Eugen Zizer[1], Umesh Tharehalli[1], Nora Daiss[1], Ronan Russell[6], Lukas Perkhofer[1], Rupert Oellinger[7], Qiong Lin[8], Ninel Azoitei[1], Frank-Ulrich Weiss[9], Markus M Lerch[9,10], Stefan Liebau[11], Sarah-Fee Katz[1], André Lechel[1],Roland Rad[7], Thomas Seufferlein[1], Hans A. Kestler[5], Michael Ott[4], Amar Deep Sharma[4], Patrick C. Hermann[1,], Alexander Kleger[1,*]*

Supporting Information

Functional Genomic Screening During Somatic Cell Reprogramming Identifies DKK3 as a Roadblock of Organ Regeneration

Frank Arnold[1], Pallavi U Mahaddalkar[2], Johann M. Kraus[3], Xiaowei Zhong[4], Wendy Bergmann[5], Dharini Srinivasan[1], Johann Gout[1], Elodie Roger[1], Alica K. Beutel[1], Eugen Zizer[1], Umesh Tharehalli[1], Nora Daiss[1], Ronan Russell[6], Lukas Perkhofer[1], Rupert Oellinger[7], Qiong Lin[8], Ninel Azoitei[1], Frank-Ulrich Weiss[9], Markus M Lerch[9,10], Stefan Liebau[11], Sarah-Fee Katz[1], André Lechel[1],Roland Rad[7], Thomas Seufferlein[1], Hans A. Kestler[5], Michael Ott[4], Amar Deep Sharma[4], Patrick C. Hermann[1,], Alexander Kleger[1,*]*

Supplementary Materials

Supplementary methods

Isolation and cultivation of mouse embryonic fibroblasts

To isolate mouse embryonic fibroblasts (MEFs), female pregnant mice were sacrificed at day E13.5 post-coitum and embryos were separated. The head and internal organs were removed from embryos. Remaining embryonic tissues were digested with 0.25% trypsin / 1 mM EDTA (Millipore) at 37 °C for 3 min followed by mechanical dissociation through pipetting. Dissociated cell suspension was cultivated in 0.2% gelatin-coated dishes in Dulbecco's Modified Eagle's Medium (DMEM, Gibco® Life Technologies™) containing 10% fetal bovine serum (FBS, Sigma-Aldrich), 1% GlutaMAX, 1 mM sodium pyruvate, 0.1 mM β-Mercaptoethanol, and 1% Penicillin/Streptomycin (P/S, all Gibco® Life Technologies™).

Isolation and cultivation of mouse embryonic stem cells

Mouse embryonic stem cells (mESC) were isolated from embryos removed from female mice at day E3.5 post-coitum. Blastocysts were collected and cultivated in M16 medium (Sigma-Aldrich) in 12-well plates coated with a feeder layer of mitomycin C-inactivated MEFs. After two days, medium was replaced every 48 hours with KOSR-ES-feeder medium containing Knockout™ DMEM (KO-DMEM; Life-Technologies) supplemented with 15% Knockout

Serum Replacement (KOSR, Life Technologies), 1% P/S, 1% GlutaMAX, 1% non-essential amino acids (NEAA, Gibco® Life Technologies™), 1% Sodium Pyruvate, 1% β-Mercaptoethanol, 240 U/ml leukaemia inhibitory factor (LIF, Cell Guidance Systems), as well as 1 µM PD0325901 (Calbiochem) and 3 µM GSK3β-inhibitor CHIR99021 (Axon Medchem) (2i). After seven days of expansion, blastocysts were harvested by 0.25% Trypsin/ EDTA for 15 sec, fully dissociated in 2.5% Trypsin and subsequently cultivated in ES-feeder medium containing KO-DMEM with 15% FBS, 1% P/S, 1% GlutaMAX, 1% NEAA, 1% Sodium Pyruvate, 1% β-Mercaptoethanol and 240 U/ml LIF in 12-well plates coated with mitomycin C inactivated MEFs. The medium was changed every 48 h.

Differentiation of mouse ESCs and embryoid body (EB) formation

Mouse ESCs were seeded and cultivated in hanging drops (400 cells / 20 µl) in N2B27 medium containing Iscove's Modified Dulbecco's Medium (IMDM) and Ham's F12 medium (ratio 3:1) supplemented with 0.5x B-27TM, 0.5x N-2 (all Gibco® Life Technologies™), 1% P/S, 0.05% bovine serum albumin (BSA, Sigma Aldrich), 1% GlutaMAX, 2 mM Ascorbic acid, and 450 µM monothioglycerol (all Sigma-Aldrich) for 48 h. The drops, each containing an embryoid body (EB), were transferred to non-adherent plates with 5 ml N2B27 medium supplemented with 50 ng/ml Activin A (PreproTech). The medium of EBs was changed with fresh N2B27 medium with Activin A every second day. Cells were carefully centrifuged at 800 rpm for 2 min, and resuspended in fresh medium on a new plate.

Lentiviral production

One day prior infection 5 x 10^5 Lenti-X cells (Takara Clontech) were seeded in a 0.1% gelatin coated 100 mm petri dish. The transfection was performed using polyethylenimine (PEI, Polysciences), and a DNA mix of 10 µg target plasmid, 5.5 µg psPAX2 and 2 µg pMD2.G (both Addgene), at a DNA:PEI ratio of 1.25 in DMEM. After 6 hours of incubation of

transfected Lenti-X cells with DNA/PEI mix, medium was replaced by fresh DMEM with 10% FBS, 1% P/S. Virus particles were harvested at day 2 and 3 post-transfection and subsequently 100-fold concentrated using Lenti-X concentrator (Takara Clontech).

FACS for SSEA1 staining and sorting

Cells (on day 13 of reprogramming) were harvested and washed twice with 2% FCS (Lonza) in PBS (FACS buffer). 1 x 10^6 cells resuspended in 50 µl FACS buffer and incubated with 1 µl of undiluted mouse anti-SSEA1 antibody (Developmental Studies Hybridoma Bank) on ice for 2 h. After washing twice with FACS buffer, cells were incubated with secondary Alexa Fluor 647 goat anti-mouse IgM (1:200, Life Technologies™) for 30 min. Subsequently, cells were washed twice, resuspended in 350 µl FACS buffer containing 4',6-diamidino-2-phenylindole (DAPI, 1:10000, Sigma-Aldrich) and analyzed and sorted with BD LSRII and BD FACSAria III cell sorter, respectively. Analysis was performed using FACSDIVA version 6.1.3 and FlowJo version 10, respectively.

Cultivation of iPSC

Single SSEA1$^+$ sorted cells were seeded and cultivated in 0.2% gelatin coated 96-wells in ES-feeder medium supplemented with 1 µM PD0325901 (Calbiochem), 3 µM GSK3β-inhibitor CHIR99021 (Axon Medchem), and 0.005 mM Thiazovivin (Calbiochem). Medium was replenished every second day.

AP expression staining

AP expression staining was performed using standard protocols. Briefly, cells on 13th day of reprogramming were fixed with 4% para-formaldehyde (PFA) and 10% sucrose in Dulbecco´s phosphate buffered saline (PBS, Thermo Fisher Scientific) for 10 min at room temperature. After two rounds of washing with PBS, iPSC colonies were stained with 2%

NBT/BCIP solution (nitro blue tetrazolium chloride and 5-bromo-4-chloro-3-indolyl-phosphate) in 0.1 M Tris-HCL (pH 9.5), 0.1 M sodium chloride, 0.05 M magnesium chloride, and 1% Tween 20 (all Sigma-Aldrich) for 15 min in the dark. After additional PBS washing, AP positive colonies were quantified manually at the microscope.

Immunofluorescence staining of mESCs or iPSCs

mESCs or iPSCs were cultivated on cover slips and fixed with 4% PFA and 10% sucrose in PBS for 15 min at RT. Blocking was conducted with 3.5% normal goat serum (NGS, Jackson ImmunoResearch) and 1% fish skin gelatin (Sigma-Aldrich) for 45 minutes before incubation with the primary antibody (Supplementary Table S2) overnight at 4°C followed by 1 h incubation with secondary antibody in the dark. Finally, cells were mounted with ProLong™ Diamond Antifade Mountant containing DAPI (Life Technologies™).

Isolation of fetal and adult liver progenitor cells

Liver from embryos at day E14.5 was minced and dissociated by incubation in 0.05% of collagenase D solution (0,5 mg/ml Collagenase D (Roche) and 5 mM $CaCl_2$ in HBSS) and mechanical dissociation. After washing fetal liver progenitor cells (LPCs) twice with PBS supplemented with 2% FBS, LPCs were stained for 1h with the following antibodies: CD13-PE (BD Biosciences), TER119-PE-Cy7 (BD Biosciences), CD133-APC (eBiosciences), CD45-PE-Cy7 (eBiosciences), cKit-PE-Cy7 (eBiosciences), and DLK-FITC (MBL). Upon antibody incubation, fetal LPCs ($CD45^-$, $Ter119^-$, $cKit^-$, $CD13^+$, $CD133^+$, DLK^+) were analyzed and sorted on a BD FACS Aria II flow cytometer.

To isolate adult LPCs, liver cells from mice (6-8 weeks) were isolated through 2-step collagenase perfusion. First, liver was perfused with 0.5 mM EGTA in HBSS for 3 min, followed by 5 min perfusion with collagenase buffer (0.4 mg/ml collagenase IV (Sigma-Aldrich) and 5 mM $CaCl_2$ in HBSS.

Adult LPCs were isolated from perfused livers. Parenchymal cells (mature hepatocytes) and non-parenchymal cells (including LPCs) were separated from each other by low-speed centrifugation (50 x g for 1 minute) and dead cells were removed by centrifugation in 25% Percoll solution (GE Healthcare). After two washing steps with PBS supplemented with 2% FBS, the non-parenchymal cells (including LPCs) were incubated with the following antibodies for 1 h: CD13-PE, CD49f-PerCP-Cy5, TER119-PE-Cy7 (BD Biosciences), CD45-PE-Cy7, CD133-APC, Sca1-PE-Cy7, and cKit-PE-Cy7 (eBiosciences). Afterwards, adult LPCs ($CD45^{-}$, $TER119^{-}$, $cKit^{-}$, $Sca1^{-}$, $CD13^{+}$, $CD49f^{+}$, $CD133^{+}$) were analyzed and single cell-sorted on a BD FACS Aria II flow cytometer.

Histology, immunohistochemistry, and immunofluorescence staining

Tissue specimens were fixed in 4% PFA overnight at 4°C and embedded in paraffin (FFPE). Hematoxylin and Eosin (HE) and Picrosirius Red (SR) staining were performed on 5 µm serial sections using standard procedures. Immunohistochemical staining (IHC), and immunofluorescence staining (IF) were performed as followed. Briefly, after deparaffinization and rehydration, antigen retrieval was performed in the microwave oven for 20 min using citric-acid-based antigen unmasking solution (Vector Laboratories). After 30 min of blocking and permeabilizing with 2% BSA (Sigma-Aldrich), 5% normal goat serum (NGS, Jackson ImmunoResearch), 0.4% Triton-X (Sigma-Aldrich) in Tris-buffered saline (TBS), tissue sections were incubated with the primary antibodies (outlined in Supplementary Table S2) overnight at 4 °C, followed by incubation with secondary antibody for 30 min at room temperature. Tissue sections for IF were immediately mounted with ProLong™ Diamond Antifade Mountant containing DAPI (Life Technologies™). Tissue sections for IHC were developed using VECTASTAIN® Elite® ABC and VECTOR® NovaRED® Peroxidase (HRP) Substrate Kit. After hematoxylin counterstaining and dehydration, tissue sections were mounted with Entellan® (Sigma-Aldrich).

RNA extraction, cDNA synthesis, and RT-PCR

RNA from powdered tissue was extracted using RNeasy Plus Mini Kit (Qiagen) according to manufacturer's protocol. RNA quantity was determined using NanoDrop. cDNA synthesis was performed using Superscript™ II Reverse Transcriptase (Thermo Fisher Scientific). Briefly, 250 ng RNA was transcribed following the user guidelines. cDNA was generated and diluted 10-fold. PCR was performed at the QuantStudio™ 3 System using 4 µl diluted cDNA together with 5 µl PowerUp™ SYBRTM Green Master Mix (Thermo Fisher Scientific), 1 µM Forward (0.5 µl) and 1 µM Reverse Primer (0.5 µl). The used primers are outlined in Supplementary Table S3. mRNA levels were normalized to housekeeping gene *Hmbs* or ribosomal protein *S18*.

Western blot analysis

Powdered tissue was lysed in 4% SDS in Tris-HCL pH 7.6 (2 ml 20% SDS, 1 ml 1M Tris-HCL pH 7.6, 7 ml sterile H2O) supplemented with cOmplete™, EDTA-free Protease Inhibitor Cocktail and PhosSTOP™ (Roche) followed by mechanical homogenization using 20G needle and incubation for 10 min at room temperature. Lysates were centrifuged and protein concentration from clear supernatant was determined using Bradford method.

15 µg protein and PageRuler™ Plus Prestained Protein Ladder (Thermo Fisher Scientific) were loaded and separated on 4-12% NuPAGE™ 4-12% Bis-Tris Protein Gels (NP0336BOX, Thermo Fisher Scientific) for 1 h at 200V using MOPS buffer. Proteins were blotted on PVDF membrane with Tris-Glycine, methanol transfer buffer for 2h. Membranes were blocked in 5% BSA in Tris-buffer saline with 0.1% Tween 20 (TBST) and incubated with primary antibody rabbit anti-p-SMAD2 (Ser465/467, 138D4, 1:1000, Cell Signaling), rabbit anti-p-p44/42 MAPK (Erk1/2, Thr202/Tyr204, 197G2, 1:1000, Cell Signaling), rabbit anti-non-phospho (Active) β-Catenin (Ser33/37/Thr41, D13A1, 1:1000, Cell Signaling), rabbit

anti-sonic hedgehog (SHH, 5H4, 1:1000, Thermo Fisher Scientific), rabbit anti-GAPDH (D16H11, 1:5000, Cell Signaling) or mouse anti-vinculin overnight at 4°C. After washing in TBST membrane were incubated in HRP-linked $F(ab')_2$ fragment goat anti-mouse and donkey anti-rabbit (both 1:5000, GE Healthcare Life Sciences) for 2 h at room temperature. Western blots were developed using SuperSignal™ West Dura Chemiluminescent-Substrate (Thermo Fisher Scientific) on a Fusion SL device (VilberLourmat).

After development blots were washed and stripped with Restore™ Western Blot stripping buffer (Thermo Fisher Scientific). Following re-blocking with 5% BSA in TBST, membranes were probed with rabbit anti-SMAD2 (D43B4, 1:1000, Cell Signaling), and rabbit anti-p44/42 MAPK (Erk1/2, 137F5, 1:1000, Cell Signaling) overnight at 4°C. Secondary antibody incubation and development were performed as described above. Intensity of protein bands were quantified using Fiji (ImageJ).

Serological parameters of mouse models

Concentration or activity of liver-specific serum enzymes (ALT, AST, and TB) were determined using Reflotron test stripes (ROCHE). The activity of the pancreas-specific serum enzymes Amylase and Lipase were measured using the Amylase Activity Assay Kit (Sigma-Aldrich) and Lipase Activity Assay Kit (Sigma-Aldrich), respectively. Absorbance was measured at Tecan Infinite M200 Pro.

Hydroxyproline assay

Hydroxyproline concentration was determined using the Hydroxyproline Assay Kit (Sigma, MAK008) according manufacturer guidelines. Ten milligram tissue was homogenized in 100 µL of water. After addition of 100 µL of concentrated hydrochloric acid (HCl, 12 M), tissue was hydrolyzed at 120 °C for 3 h. After centrifugation, 20 µL of supernatant was mixed with 100 µL of the Chloramine T/Oxidation Buffer Mixture in a 96-well plate and incubated

at room temperature for 5 min, followed by further incubation for 90 min at 60 °C with 100 μL of the Diluted DMAB Reagent. Absorbance was measured at 560 nm (A560).

Supplementary figures

Suppl. Fig. S1

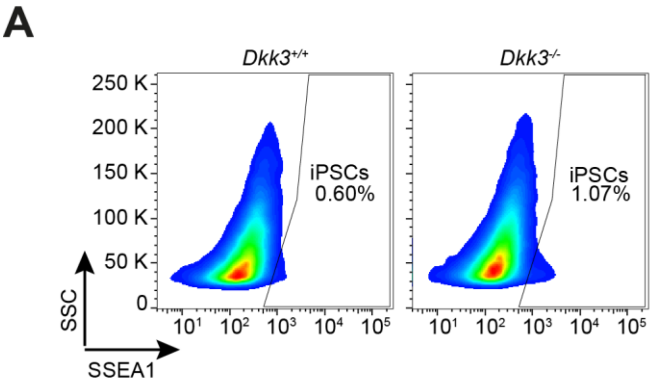

Figure S1. Dkk3 loss drives reprogramming to induced pluripotent stem cells, but does not affect de novo derivation of embryonic stem cells and three-germ layer differentiation

(A) Representative FACS-plots of SSEA1-sorting of reprogrammed *Dkk3*$^{+/+}$ and *Dkk3*$^{-/-}$ mouse embryonic fibroblasts

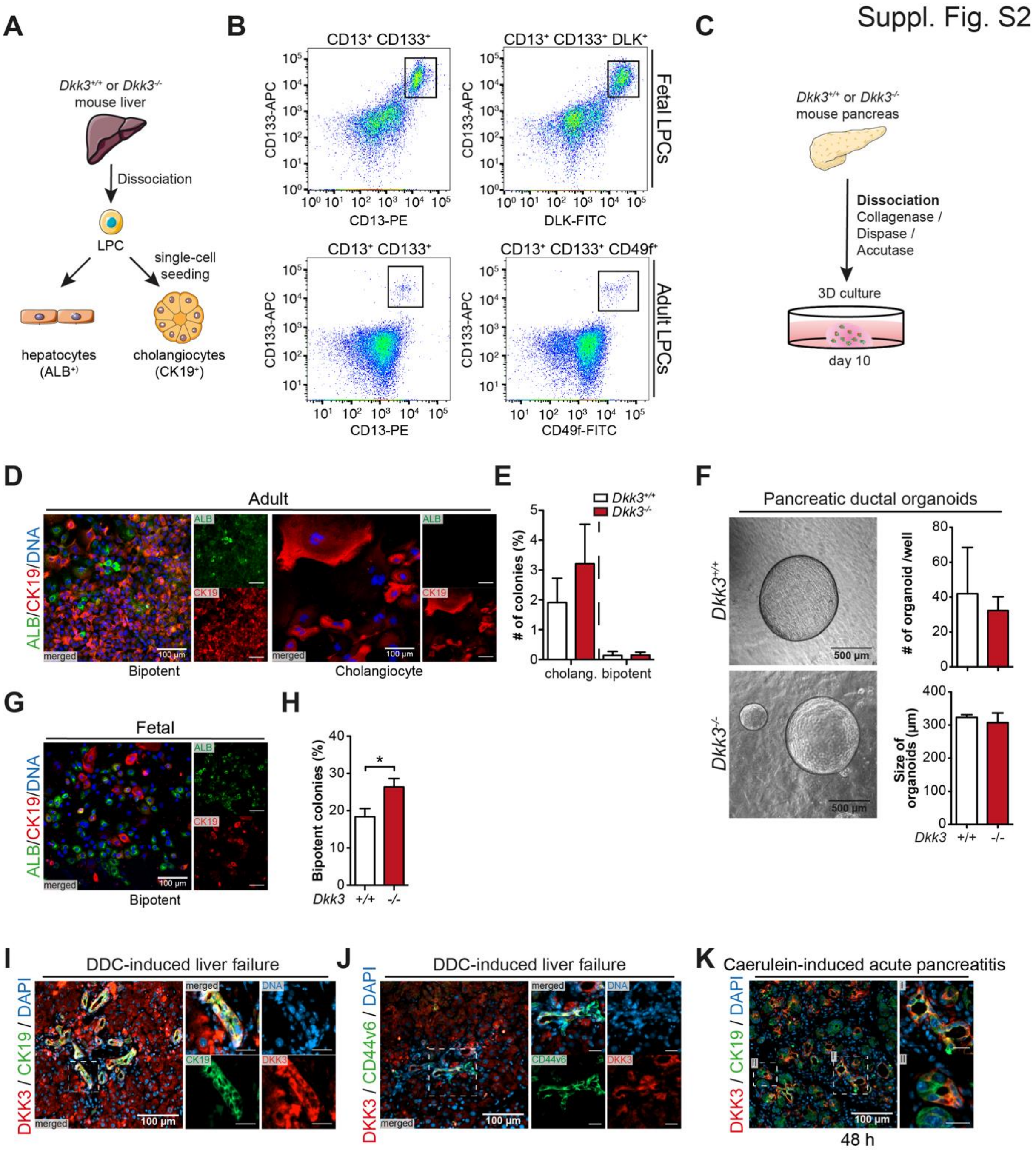

Figure S2. DKK3 loss does not impair colony forming capacity of adult liver progenitor cells or pancreatic ductal organoids

(A) Scheme showing isolation and cultivation of fetal or adult liver progenitor cells (LPC) derived from wildtype (*Dkk3*$^{+/+}$) and *Dkk3*-knockout (*Dkk3*$^{-/-}$) animals. (B) Representative FACS blots of sorting of either fetal or adult liver progenitor cells (C) Scheme showing the isolation and cultivation of pancreatic ductal organoids (PDO) derived from wildtype (*Dkk3*$^{+/+}$) and *Dkk3*-knockout (*Dkk3*$^{-/-}$) animals. (D, E) Immunofluorescence staining of

bipotent colonies (ALB^+, $CK19^+$) or cholangiocytes ($CK19^+$) from adult liver progenitor cells with (E) corresponding quantification of colony number ($n = 5$). (F) Representative pictures of PDOs at day 10 of culture with corresponding quantification of the number and size of organoids ($n \geq 3$). (G, H) Immunofluorescence staining of bipotent colonies (ALB^+, $CK19^+$) from fetal liver progenitor cells with (H) corresponding quantification of colony number ($n = 7$) (I, J) Immunofluorescence staining of (I) DKK3 and CK19 or (J) DKK3 and CD44v6 in *Dkk3*$^{+/+}$ animals with DDC-induced liver failure. (K) Immunofluorescence staining of DKK3 and CK19 in *Dkk3*$^{+/+}$ animals with caerulein-induced acute pancreatitis after 48 h. (Bar graph shows mean + SEM, unpaired t-test, * $p \leq 0.05$)

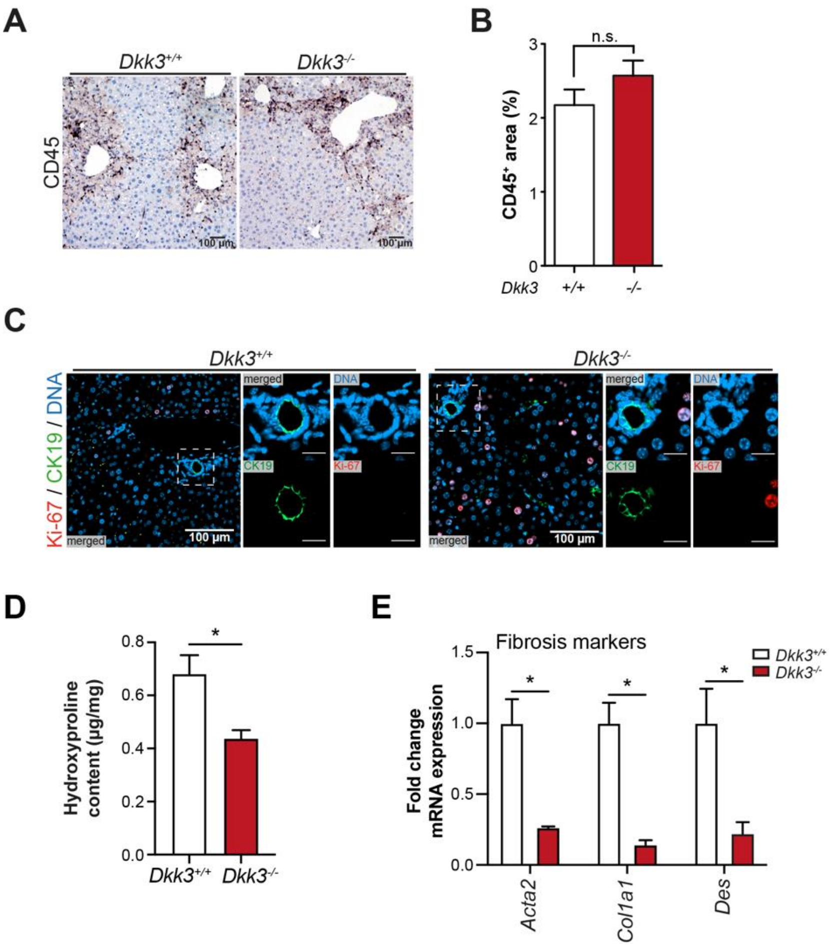

Figure S3. *Dkk3* deficiency inhibits CCl_4-induced acute and cholestasis-induced chronic liver injury.

(A) Immunohistochemistry of CD45 expression and (B) quantification of $CD45^+$ area in *Dkk3*$^{+/+}$ and *Dkk3*$^{-/-}$ animals (n = 5). (C) Immunofluorescence staining of Ki-67 and CK19 in *Dkk3* knockout and control mice 48 h after CCl4-induction. (D) Analyses of the total collagen content by the hydroxyproline assay in *Dkk3*-knockout and control mice after DDC diet for 6 weeks (n = 3). (E) RT-PCR analyses of fibrosis-related genes such as *Acta2*, *Col1a1*, and *Desmin* in *Dkk3*$^{+/+}$ and *Dkk3*$^{-/-}$ after DDC diet for 6 weeks. (n = 4); (Bar graph indicates mean + SEM, unpaired t-test, * $p \leq 0.05$)

Suppl. Fig. S4

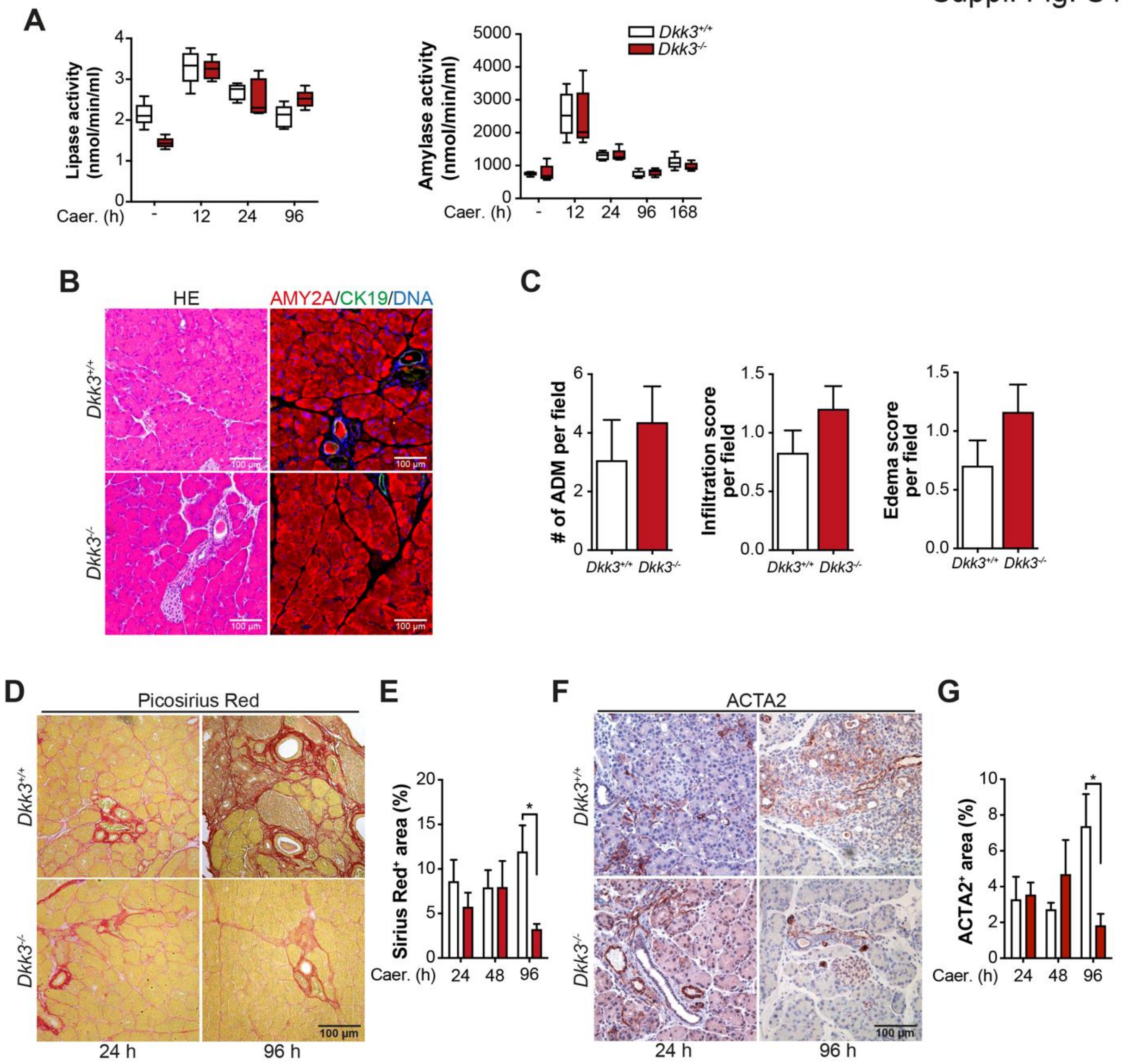

Figure S4. Loss of *Dkk3* accelerates regeneration of caerulein-induced acute pancreatitis

(A) Serological parameters of pancreas specific enzymes (Lipase, Amylase) at indicated time points of acute pancreatitis (n = 5) (B) Representative pictures of HE, and Amylase (AMY2A), CK19 immunofluorescence staining in *Dkk3*-knockout and control animals 7 days post caerulein injection. (C) Quantification of acinar to ductal metaplasia (ADM) lesions, edema, and infiltration score (D, E) Representative images of Picrosirius Red staining with quantification (F, G) Representative images of ACTA2 staining with corresponding quantification. ($n \geq 4$, bar graph shows median with min, max, unpaired t-test, ** $p < 0.01$, *** $p < 0.001$)

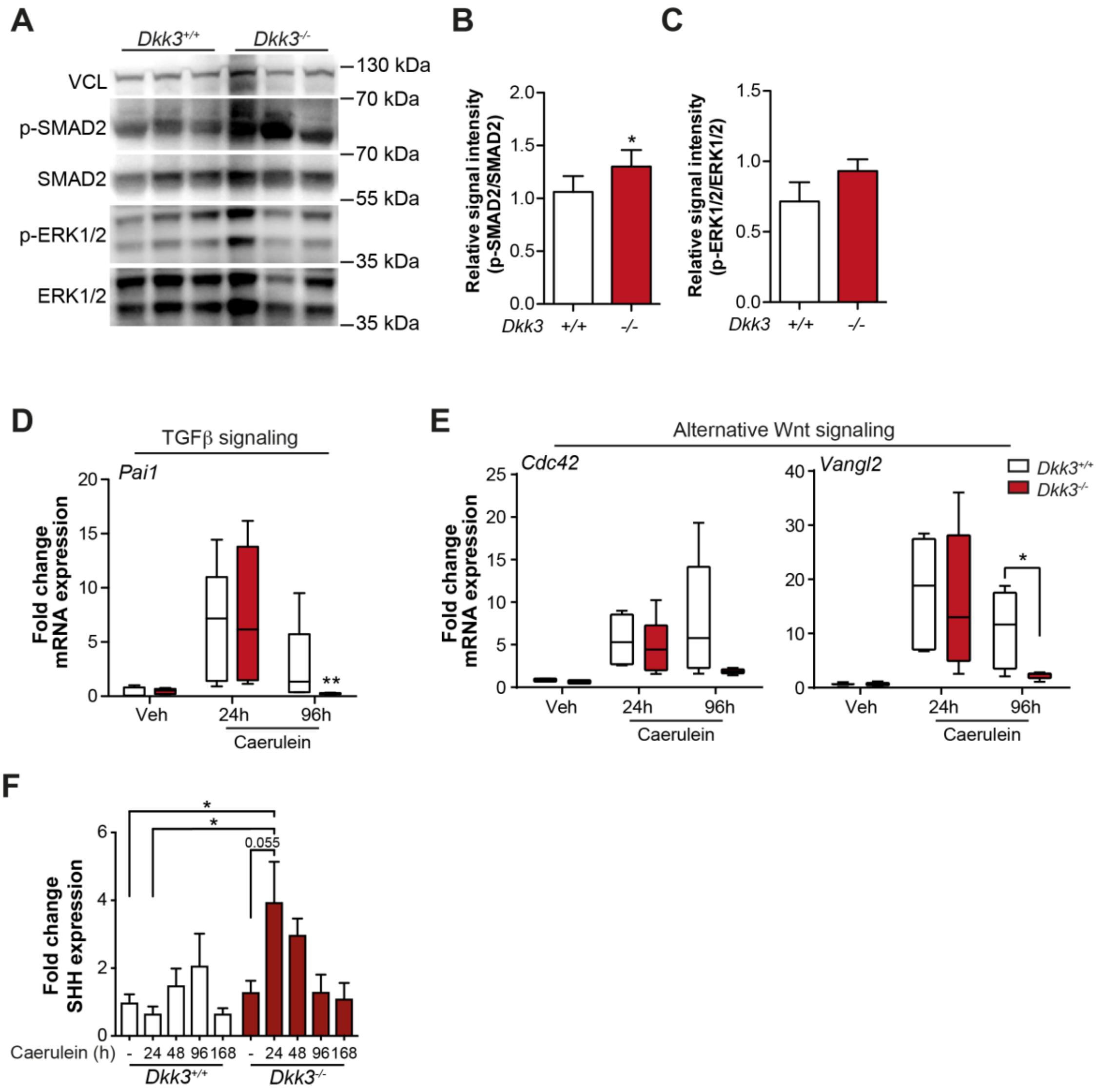

Figure S5. *DKK3* loss does not affect TGF-β-, ERK-, and non-canonical Wnt-signaling

(A) Western blot analysis of TGF-β-signaling by pSMAD2, SMAD2, and ERK-signaling by p-ERK1/2, ERK1/2 24 h after caerulein-treatment in *Dkk3*$^{+/+}$ and *Dkk3*$^{-/-}$ animals. (B, C) Quantification of intensity ratio of pSMAD2 / SMAD2 and p-ERK1/2 / ERK1/2 (n = 5). (D) RT-PCR analysis of TGF-β-signaling target gene *Pai1* and (E) non-canonical Wnt-signaling target genes *Cdc42* and *Vangl2* (n = 5). (F) Quantification of relative expression levels of Western blot analysis of sonic hedgehog at indicated time points (n ≥ 4). (Bar graphs show means + SEM, box plot shows median with min, max, statistics unpaired t-test for Western blot quantification, Mann-Whitney U test for RT-PCR, * $p \leq 0.05$, ** $p < 0.01$)

Supplementary tables

Table S1. Patient characteristics

	Control group (n = 11)	Acute pancreatitis (n = 27)	Chronic pancreatitis (n = 20)		Acute liver failure (n = 10)	Chronic liver diseases (n = 20)
Age, y, median (range)	31.1 (28.5 - 59.3)	56.5 (36.2 - 80.0)	55.6 (21.7 - 82.9)		41.6 (19.4 - 69.7)	61.8 (43.8 - 76.7)
**Sex:**						
Male, n (%)	5 (45.5)	27 (100)	13 (65.0)		6 (60.0)	13 (65.0)
Female, n (%)	6 (55.5)	0 (0)	7 (35.0)		4 (40.0)	7 (35.0)
**BMI: (Kg/m2)**						
Underweight, <18.5); n (%)	0 (0)	2 (7.4)	-		-	-
Normal, 18.5 - 24.9; n (%)	10 (90.9)	5 (18.5)	-		-	-
Overweight, 25 - 29.9; n (%)	1 (9.1)	8 (29.6)	-		-	-
Obesity, >30; n (%)	0 (0)	12 (44.4)	-		-	-
**Etiology of pancreatitis**				**Etiology of liver failure**		
Alcoholic, n (%)	-	12 (44.4)	10 (50.0)	Hepatitis B virus exposure, n (%)	1 (10.0)	-
Biliary, n (%)	-	10 (37.0)	-	Drug-induced liver injury (DILI), n (%)	3 (30.0)	-
Idiopathic, n (%)	-	5 (18.5)	10 (50.0)	Alcoholic steatohepatitis, n (%)	4 (40.0)	7 (35.0)
Pancreatic exocrine insufficiency, n (%)	-	4 (14.8)	11 (55.0)	Autoimmune-associated, n (%)	2 (20.0)	3 (15.0)
Diabetes mellitus, n (%)	-	6 (22.2)	7 (35.0)	Primary biliary cholangitis (PBC), n (%)	-	2 (10.0)
				Unknown, n (%)	-	8 (40.0)
**Clinical parameters**				**Clinical parameters**		
Lipase (U/L, median, range)	-	46.5 (1.41 - 958.0)		ALT (U/L, median, range)	472.0 (32.0 - 2004.0)	33.5 (13.0 - 129.0)
C- reactive peptide (mg/L, median, range)	-	84.0 (3.3 - 315.0)		AST (U/L, median, range)	266.0 (81.0 - 1175.0)	57.0 (21.0 - 132.0)
Leukocyte count (G/L, median, range)	-	11.4 (4.3 - 24.6)		GGT (U/L, median, range)	221.0 (96.0 - 871.0)	246.5 (22.0 - 679.0)
				Bili (µmol/L, median, range)	312.5 (8.0 - 544.0)	23.0 (7.0 - 451.0)
				AP (U/L, median, range)	142.0 (80.0 - 429.0)	133.0 (65.0 - 397.0)
				Quick (%, median, range)	59.0 (38.0 - 103.0)	62.0 (29.6 - 116.0)
				Thrombocytes (G/L, median, range)	185.5 (38.0 - 618.0)	113.5 (17.0 - 378.0)

Table S2. Used antibodies and dyes in IF and IHC

Antibody	Species	Dilution	Application	Company
**Primary antibodies**				
OCT3/4	mouse	1:100	IF	Santa Cruz
NANOG	rabbit	1:50	IF	Abcam
Albumin	rabbit	1:200	IF	Bethyl Diagnostics
CK19	goat	1:50	IF	Santa Cruz
DKK3	rabbit	1:100	IHC	Thermo Fisher Scientific
DKK3	rabbit	1:100	IHC, FFPE-IF	Thermo Fisher Scientific
Ki-67	rabbit	1:250	IHC	Thermo Fisher Scientific
Ki-67	rat	1:100	FFPE-IF	Thermo Fisher Scientific
HNF4a	mouse	1:100	FFPE-IF	Thermo Fisher Scientific
CD44v6	rat	1:100	FFPE-IF	Affymetrix eBioscience
AMY	rabbit	1:100	FFPE-IF	Sigma-Aldrich
CK19	rat	1:35	FFPE-IF	Developmental Studies Hybridoma
SOX9	rabbit	1:500	FFPE-IF	Merck Millipore
ACTA2	rabbit	1:300	IHC	Abcam
Desmin	rabbit	1:100	IHC	Abcam
CD45	rat	1:50	IHC	BD Pharmagen
CD3e	rabbit	1:150	IHC	Thermo Fisher Scientific
MPO	rabbit	1:300	IHC	Dako
F4/80	rat	1:500	IHC	Acris
**Secondary antibodies**				
anti-rabbit AlexaFluor568	donkey	1:250	IF	Life Technologies^{TM}
anti-rat AlexaFluor 488	donkey	1:250	IF	Life Technologies^{TM}
anti-mouse AlexaFluor488	goat	1:250	IF	Life Technologies^{TM}
anti-mouse AlexaFluor568	goat	1:250	IF	Life Technologies^{TM}
anti-goat Cy3	donkey	1:300	IF	Jackson Immuno Research
biotinylated anti-rat	goat	1:100	IHC	Vector Laboratories
biotinylated anti-rabbit	goat	1:100	IHC	Vector Laboratories
**Dyes**				
Direct Red (Sirius Red)			Sirius Red	Sigma-Aldrich
Eosin			HE	Sigma-Aldrich
Weigert`s Hematoxilin			Sirius Red	Roth
Mayer`s Hematoxilin			HE, ICH	Sigma-Aldrich

Table S3. Primer used in RT-PCR in this study

Gene	Fwd-5'	Rev-5'	Company
Housekeeping			
Actb	GTCCCTCACCCTCCCAAAAG	GCTGCCTCAACACCTCAACCC	Biomers
S18	GTAACCCGTTGAACCCCATT	CCATCCAATCGGTAGTAGCG	Biomers
Stem cell genes			
Sox2	Mm_Sox2_1_SG #QT00249347		Qiagen
Nanog	Mm_Nanog_3_SG #QT01743679		Qiagen
Oct3/4	Mm_Pou5f1_1_SG #QT00109186		Qiagen
Germ-layer genes			
Eomes	Mm_Eomes_1_SG #QT01074332		Qiagen
Gsc	Mm_Gsc_1_SG #QT00095221		Qiagen
Foxa2	Mm_Foxa2_1_SG #QT00242809		Qiagen
Sox17	Mm_Sox17_1_SG #QT00160720		Qiagen
Cdx2	Mm_Cdx2_1_SG #QT00116739		Qiagen
Pax6	Mm_Pax6_1_SG #QT01052786		Qiagen
T	Mm_T_1_SG #QT00094430		Qiagen
Meso1	Mm_Tcf15_1_SG #QT00243684		Qiagen
De-Differentation			
Krt19	CCTCCCGAGATTACAACCACT	GGCGAGCATTGTCAATCTGT	Biomers
Sox9	AGCCCCGGCGGAGGAAGTCGG	GATTGCCCAGAGTGCTCGCCCG	Biomers
Fibrosis			
Acta2	GTTCAGTGGTGCCTCTGTCA	ACTGGGACGACATGGAAAAG	Biomers
Col1a1	TAGGCCATTGTGTATGCAGC	ACATGTTCAGCTTTGTGGACC	Biomers
Desmin	CCTGGAGCGCAGAATCGAAT	TGAGTCAAGTCTGAAACCTTGGA	Biomers
Tgfb1	TGGAGCAACATGTGGAACTC	GTCAGCAGCCGGTTACCA	Biomers
Inflammation			
TNFa	TGCCTATGTCTCAGCCTCTTC	GAGGCCATTTGGGAACTTCT	Biomers
Il6	KiCqStart SYBR Green Primers		Sigma-Aldrich
Cxcl1	KiCqStart SYBR Green Primers		Sigma-Aldrich
Cxcl5	KiCqStart SYBR Green Primers		Sigma-Aldrich
Ccl2	CATCCACGTGTTGGCTCA	GATCATCTTGCTGGTGAATGAGT	Biomers
Ccl5	TGCAGAGGACTCTGAGACACG	GAGTGGTGTCCGAGCCATA	Biomers
canonical WNT Signaling			
Dkk3 Exon 2	CCAGCTCTCAACTACCCTCA	CATCAGCTCCTCCACCTCTCG	Biomers
Axin2	GCCAATGGCCAAGTGTCTCT	CAGACTATGGCGGCTTTCCA	Biomers
Lgr5 (liver failure)	ACCCGCCAGTCTCCTACATC	GCATCTAGGCGCAGGGATTG	Biomers
Lgr5 (GPR49,	Mm_Gpr49_1_SG # QT00123193		Qiagen
Lef1	AGTGCAGCTATCAACCAGATCCT	TTTCCGTGCTAGTTCATAGTATTTGG	Biomers
Mmp7	GGAGATGCTCACTTTGACAAGGA	ATTCATGGGTGGCAGCAAAC	Biomers
Wnt2	AGCCCTGATGAACCTTCACAAC	TGACACTTGCATTCTTGTTTCAAG	Biomers
Wnt5a	CAACTGGCAGGACTTTCTCAA	CCTGATACAAGTGGCAGAGTTT	Biomers
non-canonical WNT signaling			
Cdc42	CCCATCGGAATATGTACCAACTG	CCAAGAGTGTATGGCTCTCCAC	Biomers
Vangl2	ACTCGGGCTATTCCTACAAGT	TGATTTATCTCCACGACTCCCAT	Biomers
Hedgehog Signaling			
Ihh	TGCATTGCTCTGTCAAGTCTG	GCTCCCCGTTCTCTAGGC	Biomers
Ptch1	CCTGCACGTTTCTAGTGTGC	AGAGCCAGGACCATGACAA	Biomers
Smo	GCAAGCTCGTGCTCTGGT	GGGCATGTAGACAGCACACA	Biomers
Gli1	AGGAATTCGTGTGCCATTG	TCCGACAGCCTTCAAACG	Biomers
Gli3	CTGCGGTATCTCCTCTCATAGG	CAGCACTGTGAAGTCTACACCTG	Biomers
